# Synergism of type 1 metabotropic and ionotropic glutamate receptors in cerebellar molecular layer interneurons in vivo

**DOI:** 10.7554/eLife.56839

**Published:** 2020-05-13

**Authors:** Jin Bao, Michael Graupner, Guadalupe Astorga, Thibault Collin, Abdelali Jalil, Dwi Wahyu Indriati, Jonathan Bradley, Ryuichi Shigemoto, Isabel Llano

**Affiliations:** 1 Université de Paris, CNRS, SPPIN - Saints-Pères Paris Institute for the NeurosciencesParisFrance; 2 The Brain Cognition and Brain Disease Institute (BCBDI), Shenzhen Institutes of Advanced Technology, Chinese Academy of Sciences; Shenzhen-Hong Kong Institute of Brain Science-Shenzhen Fundamental Research InstitutionsShenzhenChina; 3 Division of Cerebral Structure, National Institute for Physiological Sciences, The Graduate University for Advanced Studies (Sokendai)OkazakiJapan; 4 Institut de Biologie de l’Ecole Normale Superieure (IBENS), Ecole Normale Superieure, CNRS, INSERM, PSL Research UniversityParisFrance; 5 IST AustriaKlosterneuburgAustria; Oregon Health and Science UniversityUnited States; RIKENJapan

**Keywords:** cerebellum, mglurs, glutamate receptors, parallel fiber, calcium imaging, in vivo, Mouse

## Abstract

Type 1 metabotropic glutamate receptors (mGluR1s) are key elements in neuronal signaling. While their function is well documented in slices, requirements for their activation in vivo are poorly understood. We examine this question in adult mice in vivo using 2-photon imaging of cerebellar molecular layer interneurons (MLIs) expressing GCaMP. In anesthetized mice, parallel fiber activation evokes beam-like Ca_i_ rises in postsynaptic MLIs which depend on co-activation of mGluR1s and ionotropic glutamate receptors (iGluRs). In awake mice, blocking mGluR1 decreases Ca_i_ rises associated with locomotion. In vitro studies and freeze-fracture electron microscopy show that the iGluR-mGluR1 interaction is synergistic and favored by close association of the two classes of receptors. Altogether our results suggest that mGluR1s, acting in synergy with iGluRs, potently contribute to processing cerebellar neuronal signaling under physiological conditions.

## Introduction

A considerable body of data, spanning from genomics and structural studies of receptors to functional studies at the synaptic level, indicates that glutamate released at excitatory synapses of the mammalian nervous system binds to both ionotropic (iGluRs) and type 1 metabotropic receptors (mGluR1s) and engages a complex pattern of signaling pathways often involving a synergistic action of both receptors (reviewed in Reiner and Levitz, 2018). In the cerebellar cortex, glutamate released by parallel fibers (PFs) binds to receptors on Purkinje cells (PCs) and on molecular layer interneurons (MLIs), which form an interconnected circuit governing the output of the cerebellar cortex. Patterns of PF activity convey information on the sensorimotor state of the animal (review by Jörntell, 2017) and determine the recruitment of MLIs through PF-MLI synapses (Bao et al., 2010). From work in brain slices it is known that several types of iGluRs (Carter and Regehr, 2000) as well as mGluR1s (Karakossian and Otis, 2004; Collin et al., 2009) are activated in MLIs by synaptically released glutamate. Furthermore, it is well documented that the PF-MLI synapse is susceptible to activity-dependent modulation such that postsynaptic AMPARs can change their Ca^2+^ permeability in response to neuronal activity (Liu and Cull-Candy, 2000). This plastic shift in Ca^2+^ permeability has been linked to specific behavioral states (Liu et al., 2010) and it is thought to involve activation of mGluR1 (Kelly et al., 2009). As MLIs are key elements of the cerebellar circuit during motor behavior and cerebellar-dependent motor learning (Wulff et al., 2009; Jelitai et al., 2016; Gaffield and Christie, 2017; Rowan et al., 2018), examining mGluR1-mediated plasticity at PF-MLI synapses is of great importance in understanding cerebellar function.

In contrast to the wealth of knowledge from slice work, the contributions of iGluRs and mGluR1s to signaling in vivo have not been investigated in any detail. Yet significant differences between work in slices and in vivo may arise from morphological perturbations due to tissue slicing (Bak et al., 1980; Dzhala et al., 2012) and from changes in either glutamate homeostasis or in molecules involved in ionotropic and/or metabotropic glutamate signaling. To fill this knowledge gap, we analyze in the present work the role of mGluR1s in generating PF-evoked Ca_i_ signals in MLIs.

When investigating Ca_i_ rises induced by beam-activation of PFs in anesthetized mice, we ask:

1. Which conditions of PF stimulation lead to robust mGluR1 signaling?
2. Is there a synergy between iGluRs and mGluR1s at the PF-MLI synapse?

Using freeze-fracture electron microscopy, we ask:

1. What is the spatial distribution of AMPARs and mGluR1s at the PF-MLI synapse and what is its functional relevance in terms of mGluR1-iGluR interaction?

In awake mice, we ask:

1. Are mGluR1s activated during an ordinary motor task such as walking?

When considered together, our results strongly suggest that mGluR1s potently contribute to cerebellar signaling under physiological conditions and furthermore, shed light on the nature of interactions between mGluR1- and iGluR-mediated signals.

## Results

### Activation of mGluR1s by repetitive PF stimulation

**Figure 1.**
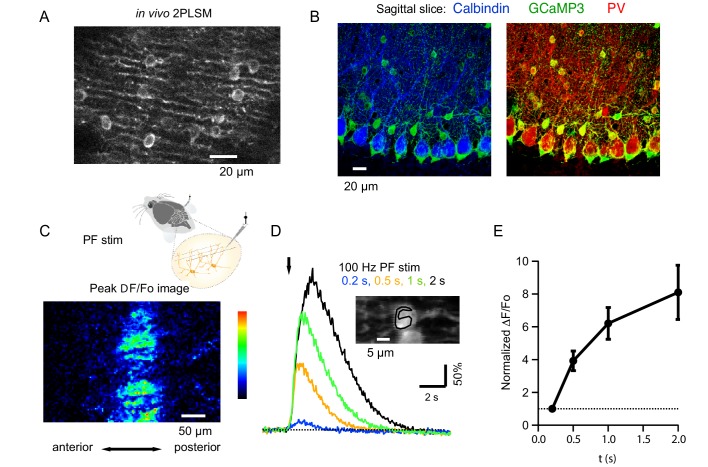
Beam activation in the cerebellar cortex of GCaMP3 expressing MLIs in vivo. (**A**) Two-photon laser scanning microscopy (2PLSM) image of the outermost cerebellar molecular layer in vivo shows cytoplasmic GCaMP3 and parasagittally aligned MLI neurites in a horizontal plane of focus. (**B**) Confocal stack projections of sagittal cerebellar slices from mice expressing AAV2/1hSyn.Flex.GCaMP3 on the background of PV-promoter driven CRE recombinase. *Left*: slice stained with an antibody against calbindin, a protein expressed in PCs and not in MLIs. Note that calbindin positive PCs do not express GCaMP3. *Right*: slice stained with an antibody against PV, a protein expressed in both PCs and MLIs. Note the colocalization between GCaMP3 and PV positive MLIs. (**C**) A beam pattern of Ca_i_ rise is illustrated by the ΔF/Fo image at the peak of the fluorescence increase evoked in the molecular layer by a 0.5 s long PF stimulation at 100 Hz (horizontal plane of focus) using a bi-polar theta glass pipette as depicted in the cartoon. The highest value for the pseudo-color scale is 100%. (**D**) Time course of the somatic MLI Ca_i_ signals evoked in a representative MLI by 100 Hz PF stimulations of various durations. The arrow indicates the time of stimulation onset. The inset shows an average of pre-stimulus images to illustrate the ROI analyzed. (**E**) Average peak ΔF/Fo values from 18 somata, normalized to the peak value for 200 ms trains at 100 Hz.

In order to assess MLI activity in vivo, we expressed the Genetically Encoded Calcium Indicators (GECIs) GCaMP3 or GCaMP5 by stereotaxic injections of floxed AAV viral vectors into the cerebellar cortex of adult parvalbumin (PV) Cre mice (*Pvalb-Cre*; see Materials and methods). Although both MLIs and Purkinje cells (PCs) are PV positive, the combined use of specific viral serotype (either AAV2/1 or AAV2/9) and of the human synapsin promoter to drive expression, resulted in the almost exclusive GECI expression in somata and neurites of MLIs (Figure 1A; see also Kuhn et al., 2012; Astorga et al., 2015). In a horizontal plane of focus, we observed a periodic narrow striped fluorescence pattern characteristic of the parasagittal organization of MLI neurites while the wider interspaced non-fluorescent stripes (about 10 μm thick) correspond to unlabeled PC dendrites. The absence of GCaMP expression in PCs is further illustrated in Figure 1B (left panel), which shows no overlap between GCaMP3 and calbindin (CB), a protein that is absent from MLIs but has a strong expression in PCs. On the other hand, the proportion of PV(+), CB(-) MLIs was very high (Figure 1B, right panel) with over 90% of MLIs expressing GECI as early as 7 days after injection. The level of expression increased during the first 2 weeks post-injection, as shown in Figure 1—figure supplement 1. The rapid time course of expression, and the MLI specificity obtained, provide us with a highly favorable situation for MLI study (Astorga et al., 2015; Astorga et al., 2017).

**Figure 1—figure supplement 1.**
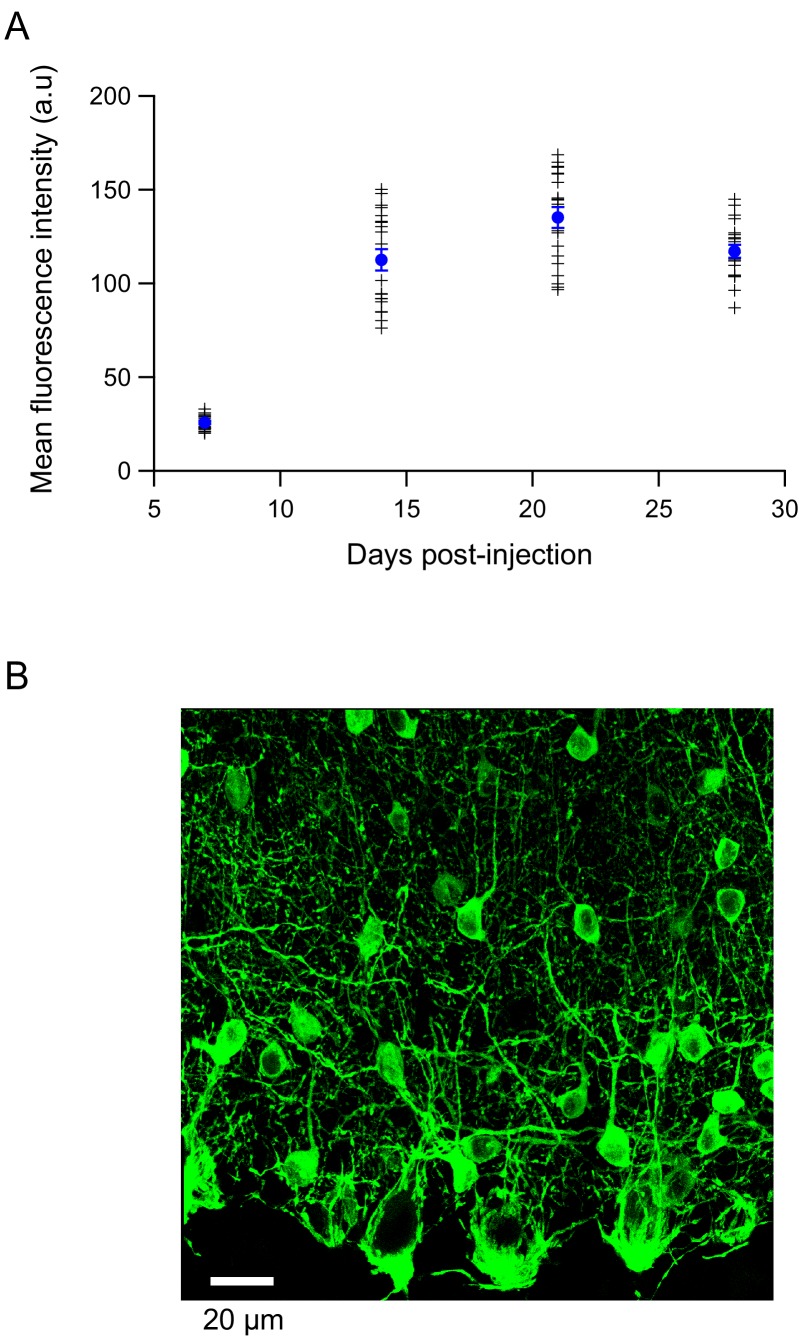
Expression of various GCaMP proteins in the cerebellar cortex. (**A**). The time course of GCaMP3 expression in the cerebellar cortex of adult *Pvalb-Cre* mice was followed in slices processed for confocal microscopy. Two mice were processed for each time point. The mean fluorescence intensity of individual MLIs somata is given by + symbols. Blue circles: mean ± s.e.m. Confocal excitation and detection parameters were kept constant for all measurements. Symbols correspond to individual somata, and the filled circle corresponds to the mean. (**B**). Using the same hsyn promoter as for GCaMP3, the expression of GCaMP5 is also restricted to MLIs, as seen in the confocal stack for a sagittal slice from a PN 45 mouse.

To activate a narrow bundle of PFs, a theta glass pipette was introduced through the meninges until it reached the outermost molecular layer (Figure 1C). In order to avoid direct stimulation of the MLIs under study, the minimal distance between the area imaged and the pipette was 150 μm. High frequency PF stimulation generated robust Ca_i_ rises in MLIs expressing GCaMP3 proteins and ΔF/Fo images displayed a characteristic beam-shaped increase in MLI fluorescence along the PF orientation (Sullivan et al., 2005; Figure 1C). Increasing the train duration led to progressively larger peak Ca_i_ transients, with ΔF/Fo values increasing from 138.9 ± 30.6% for trains lasting 0.2 s to 347 ± 43% for 1 s long trains (N = 18). An example for a representative MLI soma is displayed in Figure 1D and group data from 8 animals in Figure 1E.

**Figure 2.**
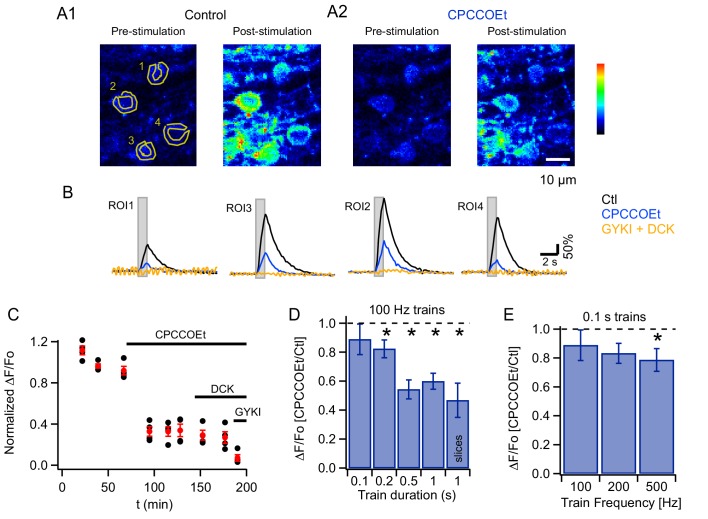
mGluR1s contribute to PF-evoked MLI somatic Cai rises. (**A**) Pharmacology of MLI Ca_i_ rises reported by GCaMP3 following PF stimulation. 2PLSM images at rest and at the peak of the response to PF stimulation (100 Hz for 1 s) in vivo in control (A1) and in CPCCOEt (A2). The pseudocolor scale in the images is expressed in Hz, calculated from the number of photons acquired during 10 μs intervals. The highest counts are 2.1 MHz. Panel **B** shows ΔF/Fo traces for the 4 ROIs drawn in the pre-stimulus image, in control (black), in 100 μM CPCCOEt (blue) and after further addition of 300 μM GYKI53655 and 100 μM DCK (yellow). (**C**) Temporal evolution of the peak Ca_i_ rise for the experiment shown in B (black: individual cells; red: mean ± sem). (**D**) Ratios of peak ΔF/Fo amplitudes in CPCCOEt over control for 100 ms (n = 20), 200 ms (n = 18), 500 ms (n = 16) and 1 s (n = 21) trains of stimuli at 100 Hz in vivo and for 1 s stimulation at 100 Hz in vitro (n = 9). Two-tailed *p* values from Wilcoxon tests are: 0.03, 9.2 × 10^−5^, 4.8 × 10^−6^ for 200 ms, 500 ms and 1 s trains in vivo and 0.008 for 1 s trains in vitro. Data from GCaMP3 and GCaMP5 expressing mice. (**E**) Ratios of ΔF/Fo peak amplitudes in CPCCOEt over control for 100 ms trains applied at different frequencies (n = 20; two-tailed *p* value from Wilcoxon test for 500 Hz: 0.04). Data from GCaMP5 expressing mice. The 100 Hz, 0.1 s bin is derived from the same data set as that used for the first bin in panel D.

To evaluate whether PF stimulation recruits mGluR1s in adult MLIs in vivo, as reported in slices for PCs (Finch and Augustine, 1998; Takechi et al., 1998) and MLIs (Karakossian and Otis, 2004; Collin et al., 2009), we compared Ca_i_ signals elicited by trains of PF stimulation (200 µs-long pulses at 100 Hz) before and after addition of a specific mGluR1 antagonist, CPCCOEt, to the pool bathing the craniotomy. We found that CPCCOEt significantly decreased peak amplitudes for the somatic signals in vivo (Figure 2A1, A2 and B3). The stability of the normalized ΔF/Fo values versus time plot in the control period, followed by a drastic drop after drug application, indicates that the decrease in Ca_i_ rises is due to mGluR1 block rather than to a run-down effect (Figure 2C). The remaining Ca_i_ signal was abolished in all cases by adding a combination of non-competitive antagonists of AMPARs and NMDARs (GYKI53655 and DCK respectively; Figure 2B, yellow traces), indicating the activation of iGluRs in MLIs. The finding of a complete block of the response by iGluR antagonists ensures that the theta stimulation does not induce direct MLI activation. In two of the experiments DCK was added prior to GYKI53655; this failed to affect the CPCCOEt-insensitive Ca_i_ rises (example in Figure 2C), indicating that this residual signal depended on AMPARs but not on NMDARs. On average, ratios of peak ΔF/Fo values in CPCCOEt over control in response to 1 s-long 100 Hz trains in vivo were similar to what we found in parallel experiments in slices (compare last two bars in Figure 2D; control peak ΔF/Fo was 347 ± 43% in vivo, n = 18; and 653 ± 58% in slices, n = 9), arguing for a strong contribution of mGluR1s in both cases.

Analysis of in vivo Ca_i_ transients in the neuropil showed a significant reduction of peak Ca_i_ values by the mGluR1 antagonist, that was however smaller than the corresponding somatic reduction (ratio of ΔF/Fo in CPCCOEt to control values: 0.73 ± 0.03 in neuropil vs 0.47 ± 0,10 in somata; two-tailed p value from Wilcoxon test: 2.1 × 10^−7^). The smaller contribution of mGluR1s to the neuropil compared to the somatic signals may reflect the fact that when analyzing the neuropil we have no unambiguous way to discriminate dendrites versus axon. Therefore, our neuropil analysis reflects a mixed population of neurites, with dendrites having a presumably more robust mGluR1 signaling than axonal processes. Nonetheless, analysis of the neuropil allows us to confirm that mGluRs are engaged in dendrites, where the density of parallel fibers synapses is 3 fold higher than in the soma (Abrahamsson et al., 2012).

Focusing again on somatic responses, we then examined the time window necessary for efficient mGluR1 recruitment in vivo, by quantifying the block by CPCCOEt for trains of different durations while keeping the stimulus frequency at 100 Hz. We found that as the train duration was shortened the extent of block decreased, such that with a 100 ms train essentially no mGluR1 component was observed (pooled data from 8 animals in Figure 2D).

We next asked whether increasing stimulation frequency enhances mGluR1 responses during short bursts of activity. This series of experiments was performed with GCaMP5, which yielded similar expression pattern as GCaMP3 (Figure 1—figure supplement 1) while offering a better dynamic range and improved signal-to-noise ratio (Akerboom et al., 2012). Signals elicited by 100 ms-long trains at frequencies of 100, 200 and 500 Hz were compared before and after addition of mGluR1 blocker. Pooled data analysis (Figure 2E) indicated that a significant mGluR1 component was present only for the highest frequency tested (500 Hz). As before the signals were abolished by a combination of GYKI53655 and DCK.

### Activation of mGluRs in MLIs during locomotion

**Figure 3.**
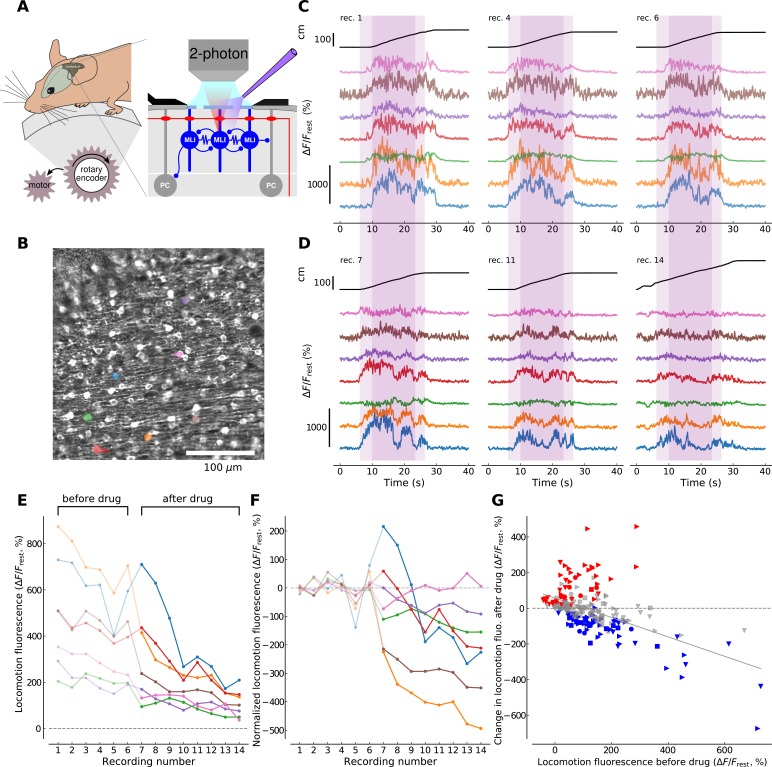
mGluR1s contribute to large-amplitude, locomotion-related Ca^2+^ activity in MLIs in the medial cerebellum. (**A**) Illustration of the forced locomotion experiment with simultaneous calcium imaging of MLIs. Mice can walk freely but the treadmill is motorized during a 20 s period, corresponding to the area shaded in purple in C and D between 6 and 26 s (dark purple corresponds to the 15 s maximal speed motorization period between 10 and 25 s of the recordings). At the right, a simplified circuit diagram of the cerebellar molecular and Purkinje cell layer illustrating craniotomy, 2 photon scanning and drug delivery. (**B**) Horizontal view of viral-mediated GCaMP3 expression in MLIs of a *Pvalb-Cre* mouse. Example ROIs are colored (see panels C,D,E,F). (**C**) Ca^2+^ responses during three non-consecutive locomotion recordings (recording number upper left; recordings repeated every 2–4 min, see Materials and methods). Top black traces show the distance traveled by the mouse during the recordings. Bottom traces show fluorescence for 7 randomly chosen example ROIs corresponding to individual cell bodies (see color-coded regions in **B**). The time period used for analysis is shaded in dark purple. (**D**) Same depiction as in (**C**) but after the application of CPCCOEt. (**E**) Calcium responses before and after CPCCOEt application. The 75^th^ percentile of the fluorescence trace during the maximal-speed locomotion period [10, 25] s, dark purple shaded region in (**C**,**D**) is shown as a function of the recording number. For this example mouse (animal #2), 6 recordings before- and 8 after drug delivery were performed. (**F**) Calcium activity normalized to pre-drug baseline. The pre-drug decay in fluorescence was subtracted from all points per ROI (see Materials and methods for details). Data for the same ROIs is shown in the same color in panels (**B**-**F**). Data points before CPCCOEt application are in pale color, data after drug delivery in dark color. (**G**) Change in fluorescence with CPCCOEt vs. pre-drug baseline data from all animals (*N*_animals_ = 4) and ROIs (*N*_ROIs_ = 313). The change in fluorescence is shown as a function of the pre-drug baseline fluorescence during locomotion. Data from each animal is plotted with the same symbols. The color of each symbol indicates whether a significant increase (red, p<0.01, T-test), decrease (blue, p<0.01, T-test) or no change (gray) has been observed when comparing baseline locomotion fluorescence with fluorescence after CPCCOEt application (see Materials and methods for more details). The linear regression on all points yields a correlation of −0.547 with p-value<0.0001, explaining 30.0% of the variance in the data.

To examine the conditions for mGluR1 activation in behaving animals, we designed a forced locomotion paradigm combining a motorized, flat-surface treadmill with 2P resonant scanner imaging in the cerebellar cortex of head-fixed mice expressing GCaMP3 (Figure 3A) and assessed the effects of mGluR1 block on the resulting Ca_i_ signals. Drug access was enabled through holes in the coverslip (<500 μm) and quantified by adding the fluorophore Alexa 594 to the drug containing solution (see Materials and methods and Figure 3—figure supplement 1). We aimed to reduce the inherent variability in awake behavioral paradigms by imposing a constant walking speed of 10 cm/s across recording sessions (see Materials and methods). 70–120 ROIs corresponding to MLI cell bodies were identified in each field of view (400 × 400 μm, see Figure 3B). Many of the identified cells exhibited fluorescence dynamics correlated to locomotion, that is, the fluorescence signal was flat and low during resting periods while it increased and showed temporal dynamics during locomotion periods (Figure 3C). We performed this experiment on 4 animals and identified in total 313 soma present in the imaging planes in both conditions, that is, before and after drug application (see Materials and methods).

**Figure 3—figure supplement 1.**
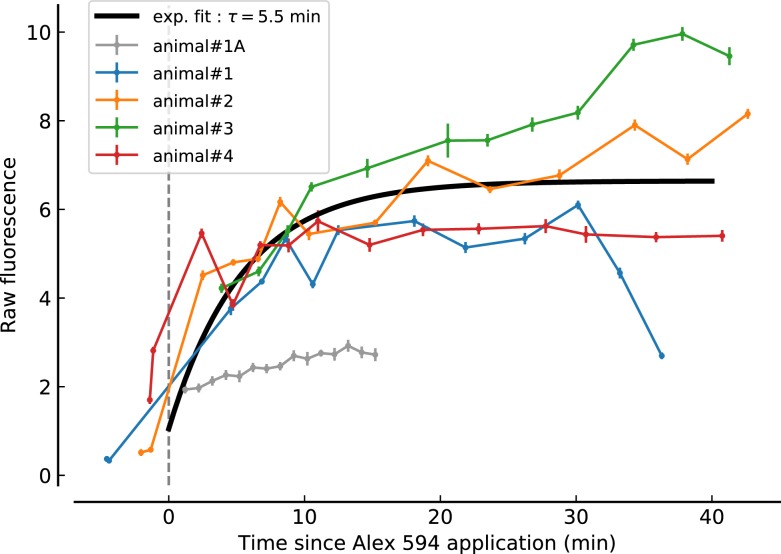
Summary of Alexa 594 fluorescence changes related to drug application for all animals. Raw Alexa 594 fluorescence is shown as function of time since application at *t =* 0 min. 50 μM Alexa was added to the drug-containing ACSF to assess the dynamics of drug diffusion in the recorded brain area. Each animal is depicted by a different color. Note that the recording of *animal#1A* was excluded from the analysis due to the inefficiency of drug application in the area of interest as reported by in impaired increases in Alexa fluorescence (gray points and line). A single exponentially increasing function (black line) was fitted to the data for t > 0 and starting at the average pre-application fluorescence average across all animals. Each recording point comprises 100 images recorded at 30 Hz of a 400 × 400 μm field of view and pixel size of 0.78 × 0.78 μm. Data-points display average and standard deviation of the image stack fluorescence values.

**Figure 3—figure supplement 2.**
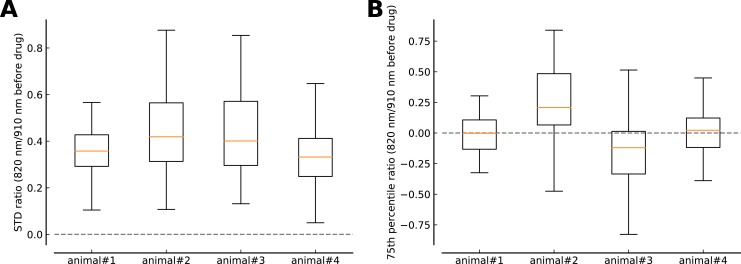
Comparison of fluorescence fluctuations and level during the recordings at 820 nm excitation wavelength with respect to the runs at 910 nm. (**A**) Ratio of fluorescence standard deviations (STD) during the locomotion period (t∈(10,25 s) between recordings at 820 and 910 nm laser excitation wavelength. Individual ratios are calculated for ROIs which have been recorded, aligned and matched (>30% overlap, see Materials and methods) between the two laser excitation conditions. The box-plots (median: orange; quartiles: box; range of data excluding outliers: whiskers) summarize the ratios for all ROIs recorded in one animal. (**B**) Same depiction as in (**A**) but for ratio of fluorescence 75^th^ percentiles during the locomotion period (t∈(10,25 s) between recordings at 820 and 910 nm laser excitation wavelength.

To directly measure the contribution of mGluR1s to the observed Ca_i_ transients, we repeated 6–14 forced locomotion recordings before and after application of CPCCOEt in four animals (compare Figure 3C and D). We then quantified the fluorescence during a 15 s period of maximal speed motorization (between 10 and 25 s in the recordings, Figure 3C,D, dark shaded areas; see Materials and methods). As we aimed at focusing on the large fluorescence increases, we extracted the 75th percentile of the ΔF/F_rest_ trace per cell during the locomotion period, hereon referred to as *locomotion fluorescence* (Figure 3E). We observed an overall reduction in locomotion fluorescence with increasing number of recordings, which was shared across all ROIs (see Figure 3E). To remove this decay trend and single out drug-induced changes in locomotion fluorescence, a single exponential decay function was fitted to all *pre-drug* data-points per individual ROI and subtracted from *all* recordings of this ROI (compare Figure 3E and F, see Materials and methods). In control conditions, ΔF/F_rest_ signals during locomotion had peak values of 128.2 ± 8.5% (313 ROIs from 4 mice). We then compared the mean level of locomotion fluorescence before drug application to the change in locomotion fluorescence after drug application (Figure 3G). Individual cells showed significant increases (red symbols in Figure 3G), decreases (blue symbols; note large reduction in example cells coded by orange, burgundy and blue in Figure 3E and F) or no significant change (gray symbols) in their after-drug locomotion fluorescence compared to before-drug recordings. Nevertheless, the direction and the amplitude of fluorescence changes appeared to be predicted by the overall level of pre-drug locomotion fluorescence. Indeed, a linear regression analysis on all the cells gave a correlation of −0.547 (R^2^ = 0.3, p<0.0001) meaning that a more probable decrease in Ca_i_ signal happened after CPCCOEt application if a higher fluorescence response was elicited during locomotion before drug application. These results suggest that under control conditions large signals are more likely to involve mGluR1s than small signals, in accord with the finding that longer or higher frequency PF stimulations are more successful in recruiting mGluR1 (Figure 2D and E). Altogether, the experiments argue for an involvement of mGluR1 in physiologically relevant situations.

### Synergy between iGluRs and mGluR1s is not mediated by Ca^2+^-permeable AMPARs

**Figure 4.**
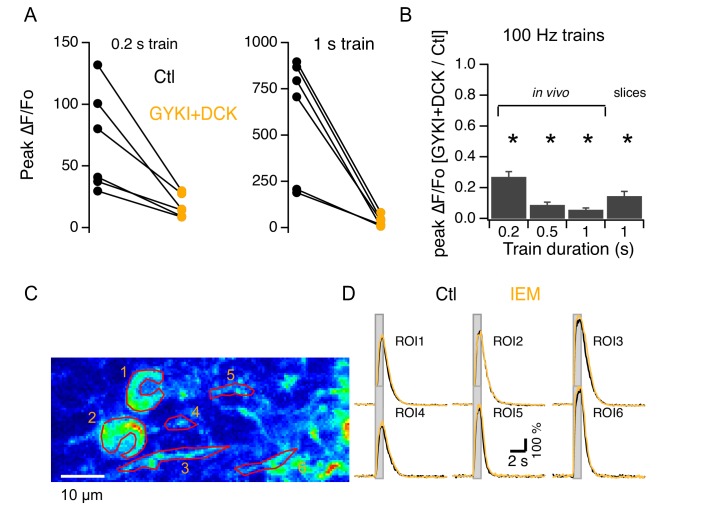
Synergy between iGluRs and mGluR1s in adult MLIs. (**A**) A mixture of 300 μM GYKI53655 and 100 μM DCK blocked somatic Ca_i_ rises under various PF stimulation protocols in vivo. (**B**) Ratios between peak amplitudes of ΔF/Fo in the blockers over control (n = 6 in each condition). (**C,D**) GCaMP3 imaging of PF-induced Ca_i_ rises in 2 somata and 4 putative neurites in vivo shows no difference between control (black traces) and addition of 40 μM IEM 1460 (yellow traces).

We next investigated whether iGluR activation was required for the mGluR1-linked Ca_i_ increases at PF-MLI synapses, as reported previously for PF-PC synapses (Okubo et al., 2004). We found that simultaneous addition of GYKI53655 and DCK (which together should abolish iGluR activation) strongly decreased PF-evoked Ca_i_ rises for all train durations tested in slices as well as in vivo (Figure 4A and B). The extent of this inhibition was much larger than the value expected from the earlier results of Figure 2 if the fractions of inhibition by ionotropic and metabotropic blockers were independent. Thus, considering that CPCCOEt blocks close to 50% of the signal elicited by a 100 Hz stimulation train lasting 1 s (ratio of drug to control: 0.47 ± 0.12; Figure 2D), the block by iGluRs would be expected to be around 50%. But the effect of iGluR block is much larger: for the same 100 Hz, 1 s long train the drugs block 94% of the response (ratio of drugs to control: 0.06 ± 0.01; Figure 4B). This suggests that while responses to short trains of PF stimulation are governed mainly by iGluRs, synergy between iGluRs and mGluR1s occurs at the PF-MLI synapse during long trains. Earlier work in slices has shown that mGluR1-dependent Ca_i_ responses in MLIs depend on Ca^2+^ entry (Collin et al., 2009). Therefore, we hypothesized that the synergy between iGluRs and mGluR1s arises either from Ca^2+^-influx through Ca^2+^-permeable iGluRs, or from voltage-gated Ca^2+^-entry following iGluR-induced depolarization.

**Figure 5.**
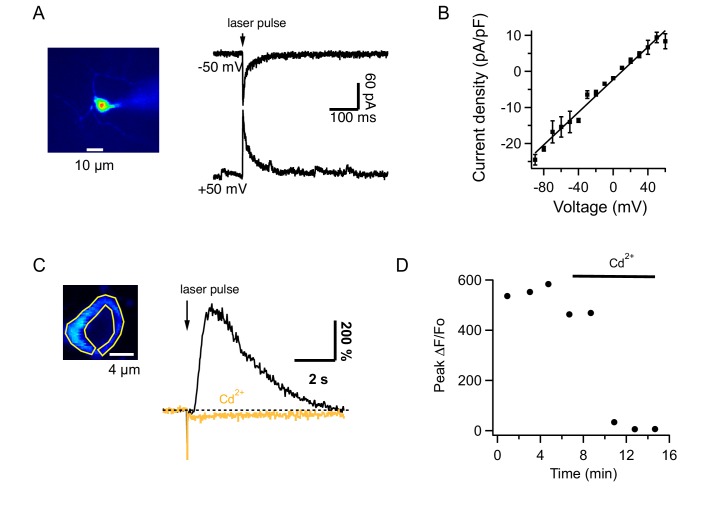
Glutamate uncaging from MLIs in slices. (**A**) MLI currents evoked by MNI-glutamate uncaging in a slice of an adult mouse at different holding potentials in the presence of 100 μM intracellular spermine. (**B**) The I-V curve from 7 MLIs shows no rectification. (**C**) *Left*: Image of an MLI expressing GCaMP6s with ROI drawn over the cytosol. *Right*: ΔF/Fo signals evoked by MNI glutamate uncaging in control saline (black) and after addition of 100 μM CdCl_2_ to the solution bathing the slice (orange). (**D**) Temporal evolution of ΔF/Fo signals for the MLI shown in C.

Under certain conditions, Ca^2+^-permeant AMPARs (CP-AMPARs) occur at the PF-MLI synapse (Cull-Candy et al., 2006; Soler-Llavina and Sabatini, 2006). We therefore tested in vivo the effect of IEM1460, which inhibits preferentially CP-AMPARs (Magazanik et al., 1997). We found that this compound had no effect on the PF-evoked Ca_i_ increases measured from the soma or the neuropil (Figure 4C and D; ratios of control to IEM: 1.2 ± 0.05 and 1.0 ± 0.06 for 200 ms and 1 s trains at 100 Hz; n = 6 somas from 2 animals), suggesting a lack of Ca^2+^-permeability for AMPARs in MLIs of adult mice. To test this possibility further, we next assessed the Ca^2+^ permeability of AMPARs in adult MLIs in slices by analyzing the I-V relations of currents evoked by uncaging of MNI glutamate (Figure 5A). All MLIs tested (n = 7) yielded linear I-V relations characteristic of Ca^2+^ impermeable AMPARs (Cull-Candy et al., 2006; Figure 5B). Therefore, the synergistic dependence on iGluRs cannot be attributed to Ca^2+^ flux through AMPARs. Overall, in contrast to findings in the juvenile cerebellum (Cull-Candy et al., 2006; Soler-Llavina and Sabatini, 2006), we did not detect CP-AMPARs in adult cerebellar MLIs either in slices or in vivo. These data indicate that AMPARs lose their Ca^2+^ permeability during development, a developmental switch already reported for glutamatergic synapses in different brain regions (Kumar et al., 2002; Eybalin et al., 2004; Isaac et al., 2007).

The remaining scenario is AMPA-mediated depolarization recruiting voltage-gated Ca^2+^ channels. To test this hypothesis, we investigated the pharmacological properties of Ca_i_ rises elicited by glutamate uncaging in slices. Signals evoked in MLIs by glutamate uncaging had peak ΔF/Fo values of 342 ± 47% (n = 12) in control saline and 5 ± 3% (n = 10) in the presence of CPCCOEt, showing that they were mediated by mGluR1. We found that these Ca_i_ rises were strongly decreased by addition of CdCl_2_ to the extracellular solution (Figure 5C and D). On average, peak ΔF/Fo decreased from 438 ± 47% to 6 ± 2% (n = 4). These results indicate that iGluRs acts on the mGluR1-induced response via voltage-gated Ca^2+^ channels.

### mGluR1 localization at PF-MLIs synapses

**Figure 6.**
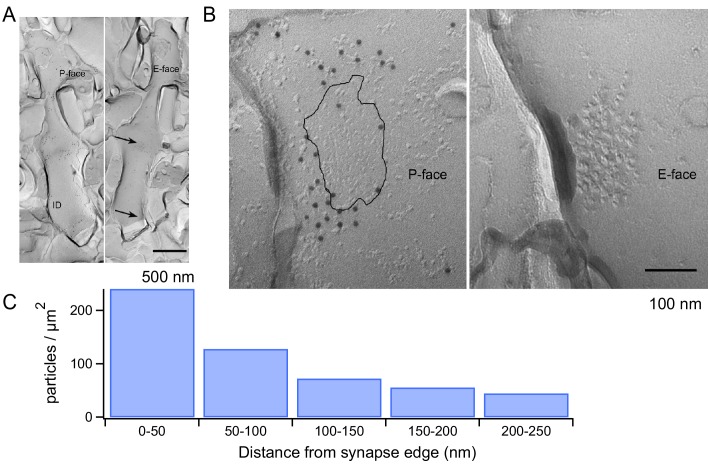
mGluR1 distribution at PF-MLI synapses. (**A**) Low magnification electron micrographs show MLI dendrites in mirror replicas with mGluR1α labeling (10 nm gold particles) on the protoplasmic face (P-face) and intra-membrane particle clusters (arrows) indicating glutamatergic postsynaptic sites on the exoplasmic face (E-face). Scale bar, 500 nm. (**B**) Immunogold labeling of freeze-fracture replicas shows mGluR1α on the P-face concentrated close to the PF-MLI postsynaptic site (black line) identified by intramembrane particle (IMP) clusters on the corresponding E-face. Scale bar, 100 nm. (**C**) Immunogold particle density as a function of distance from the synapse edge. A similar trend was found in replicas from 2 other animals, in which 241 and 191 particles were analyzed.

We next explored the structural correlates of the mGluR1 signaling with electron microscopy. Using SDS-treated freeze-fracture replica labeling, it has been shown that AMPARs form dense clusters filling the entire area of the postsynaptic density (PSD) at PF-MLI synapses (Masugi-Tokita et al., 2007). Given the dependence of mGluR1s on AMPAR activation, we used the same technique to examine the location of mGluR1s with respect to the PSD. We used an mGluR1α antibody because strong mGluR1α immunoreactivity has been described for all GABAergic neurons in the cerebellar cortex (Baude et al., 1993) while no immunoreactivity has been found in MLIs for other mGluR1 isoforms (mGluR1b and mGluR1c) (Grandes et al., 1994). The mGluR1α antibody showed labeling on the P-face of the plasma membrane, whereas no specific labeling was found on the E-face. The MLI dendrites were identified in replica samples by their smooth and thin appearance without spines (Figure 6A). The distance from PSD to each gold particle was measured from mirror replicas with mGluR1α labeling on the P-face and intramembrane particle (IMP) clusters indicating glutamatergic postsynaptic sites on the E-face (Figure 6B). The area of IMP clusters was demarcated manually and copied on the corresponding P-face area, where mGluR1α labeling was detected. From the edge of PSD, 50, 100, 150, 200 and 250 nm distance lines were drawn and the density of mGluR1α particles in these 50 nm-width areas was calculated. This analysis showed that mGluR1α are remarkably close to the PSD, with the highest density occurring within the first 50 nm from the edge of the synapse (Figure 6C). Thus, mGluR1s are ideally located to engage in synergistic interactions with AMPARs following the release of glutamate by presynaptic PFs.

## Discussion

Three main findings arise from the present work. Firstly, we show that mGluR1s are activated in vivo by synaptically-released glutamate both during electrical stimulation of PFs in anesthetized mice and during enforced locomotion in awake mice. Furthermore, we show that the relative contribution of mGluR1 activation to the postsynaptic Ca_i_ signals increases sharply as a function of the duration of presynaptic action potential trains. Secondly, we show that activation of mGluR1s and iGluRs act in synergy to produce Ca_i_ rises. Third, we show that the spatial distribution of these receptors at the PF-MLI synapse, as revealed by quantitative EM, favors iGluR/mGluR1 synergy following homosynaptic receptor activation.

### Activation of mGluR1s in vivo

Given the involvement of mGluR1s in cerebellar processes as diverse as synaptic efficacy (reviews by Knöpfel and Grandes, 2002; Kano et al., 2008; Hartmann and Konnerth, 2009), AMPAR Ca^2+^ permeability (Kelly et al., 2009), Ca^2+^ channel activity (Zheng and Raman, 2011), synapse elimination (Uesaka et al., 2014) and long term synaptic plasticity (Aiba et al., 1994; Conquet et al., 1994; Welsh et al., 2005), it is important to determine conditions governing mGluR1 activation in vivo. MLIs, the neurons of interest in the present study, have long been considered to participate in cerebellar coding through lateral inhibition (Eccles et al., 1966), with recent studies lending further support to this view (Dizon and Khodakhah, 2011). We present here the first evidence for recruitment of mGluR1s on MLIs in vivo. We further show that high frequency trains of PF activity, resembling sensory input to the cerebellar cortex and spike bursts recorded from granule cells (GCs) in vivo during somatosensory peripheral stimulation (Jörntell and Ekerot, 2006), engage mGluR1s depending on the activation of iGluRs. Previous documentation of mGluR1 activation in vivo is very scarce. While mGluR1-dependent Ca_i_ signaling has been observed before in vivo following beam activation of PFs (Wang et al., 2011), this signal may have been indirect as it had a latency >10 s; furthermore, its cellular origin remained uncertain, even though it was suggested that the primary signal source was PCs. By contrast, our work indicates direct mGluR1 activation at PF-MLI synapses with a latency well below 1 s. Demonstrating mGluR1 activation at PF-MLI synapses is particularly significant, since in contrast to PF-PC contacts, these synapses lack ensheathing elements that slow neurotransmitter concentration decay and that favor the activation of slowly activating receptors such as mGluR1s. Therefore, our results suggest that mGluR1 activation in vivo may be a widespread phenomenon, occurring under a variety of morphologically different synapses, and following common presynaptic activity patterns.

The activation of MLIs during locomotion reported here is expected as we recorded in lobule IV/V of the cerebellar vermis which encodes fore- and hindlimb sensory-motor information (Provini et al., 1968; Buisseret-Delmas and Angaut, 1993). It is in accord with recent work showing that both electrical activity (Jelitai et al., 2016) and activity-dependent Ca_i_ signals in MLIs increase during locomotion (Ozden et al., 2012). How comparable are the two experimental paradigms used in the present work ? Although peak ΔF/Fo signals are comparable (114 ± 31 and 347 ± 43% for 0.2 and 1 s long trains of electrical stimulation at 100 Hz and 128.2 ± 8.5% during locomotion), spatial and temporal patterns of activation differ. Thus, the electrical stimulation we used imposes a ‘beam’ like pattern of MLI recruitment (with a beam width in the range of 20 to 50 μm; Figure 1, see also Astorga et al., 2015). On the other hand, locomotion engages a much larger population of MLIs (Figure 3) in accord with results from Ozden et al. (2012). Concerning the number of PFs recruited, we presume that action potential thresholds are rather homogeneous across PFs when extracellular stimulation is used, so that all PFs within a beam are excited at the frequency imposed by extracellular stimulation. During locomotion, likewise, a vast majority of GCs are activated, 94% of all GCs in a large field of view, according to Ozden et al. (2012) and the impact on MLIs is a change in firing rate from an average value of 20 Hz in quiet mice to 60 Hz during self-paced locomotion (Jelitai et al., 2016). GC firing in mice walking spontaneously is very heterogeneous in time and in space. In time, firing occurs in high frequency bursts with instantaneous frequencies within a burst ranging from 60 to 142 Hz, and burst durations ranging from 30 to 140 ms. Strikingly, these values are close to those needed to obtain measurable mGluR responses with extracellular stimulation (Figure 2). In space, individual GCs are reported to vary markedly in their firing patterns when mice are walking. In view of the results of Figure 3 indicating a measurable mGluR contribution in a rather small proportion of MLIs, it is plausible that particularly active GCs cooperate within the dendritic field of these particular MLIs to produce mGluR activation as a result of temporal and spatial summation of the perisynaptic glutamate concentration.

Our finding that mGluR1 block affects the magnitude of the Ca_i_ rises associated with locomotion points to an important role of mGluR1s in cerebellar signal processing during physiologically relevant behaviors. The correlation between the extent of block and the Ca_i_ rise amplitude may arise from different patterns of PF activity during the behaviors. Together with our results on PF stimulation, the data from behaving mice aids to generate a picture of mGluR1 activation in the MLIs. During natural behavior, PFs carry sensory codes to the cerebellar cortex where sensory feedback is converted to a well programmed sequence of excitation and inhibition of PCs. MLIs provide the indispensable inhibition (Chu et al., 2012; Jelitai et al., 2016; Gaffield and Christie, 2017; Rowan et al., 2018) and the PF-MLI synapses are the sites controlling the amount of inhibition output (Bao et al., 2010). High frequency or prolonged PF activity will induce strong Ca_i_ rises in MLIs through the synergy between iGluRs and mGluR. This synergy works as a nonlinear switch, gating the plasticity at PF-MLI synapses and governing the inhibition output of MLIs.

### Synergistic Ca_i_ rises following combined activation of mGluR1s and iGluRs

In slices, the response of MLIs to mGluR1 agonists is abolished by removal of external Ca^2+^ ions (Collin et al., 2009) or by inclusion of the VGCC blocker Cd^2+^ (this work). This shows that voltage-gated Ca^2+^ entry is necessary for mGluR1-activated Ca_i_ rises. In this context, our in vivo results showing that mGluR1-dependent rises are totally dependent on simultaneous iGluR activation strongly suggest that AMPAR activation provides a depolarization that activates VGCCs, thus initiating the Ca_i_ rise and bootstrapping the mGluR1-activated response. Although most of our experiments used both AMPAR and NMDAR blockers, the evidence shown in Figure 2 indicates that AMPARs alone are responsible for the synergy. This is in accordance with the reliance of mGluR1-dependent IP3 production on AMPAR activation at PF-PC synapses (Okubo et al., 2004). A plausible source for the synergistic nature of the signals is the Ca^2+^-dependence of phospholipase C, which has been documented in cerebellar PCs (Kim et al., 2011). Taken together these results suggest that AMPAR/mGluR1 synergy constitutes a double key system controlling postsynaptic Ca_i_ responses following prolonged PF stimulation. This double key control is analogous to the well-documented joined requirement for AMPAR and NMDAR activation in the induction of long-term synaptic plasticity. Interestingly, while NMDARs are present extrasynaptically in MLIs (Carter and Regehr, 2000; Clark and Cull-Candy, 2002), the PF-MLI synapses lack NMDARs (Glitsch and Marty, 1999; Clark and Cull-Candy, 2002). Our data indicates that Ca^2+^-permeable AMPARs are also absent at this synapse. Nevertheless, PF-MLI synapses are the site of various forms of synaptic plasticity (reviewed by Dean et al., 2010). The combined activation of AMPARs and mGluR1s at PF-MLI synapses could control the rise of a postsynaptic calcium signal responsible for associative synaptic plasticity, similarly to the combined activation of AMPARs and NMDARs in other synapses. Likewise, in a subset of interneurons in the hippocampus, a form of long-term synaptic plasticity requires simultaneous activation of mGluR1s, of Ca^2+^-permeant AMPARs, and of T-type Ca^2+^ channels (Nicholson and Kullmann, 2017). Interestingly, there is another form of synergy between iGluRs and mGluRs involving the electrical field induced by activation of iGluRs, which retards the escape of charged glutamate from the synaptic cleft and favors the activation of mGluRs (Sylantyev et al., 2013). Our data does not allow us to exclude this possibility, but this phenomenon alone is unlikely to account for the observed synergy, because this synergy depends on Ca^2^ influx.

### Distribution of mGluR1s

It is interesting to compare the mGluR1α distribution on MLIs and PCs, as these two neuron types share a common presynaptic element but differ regarding the nature of the postsynaptic partner and the morphology of the postsynaptic site. PF-PC synapses are located on PC spines while PF-MLI synapses occur on smooth MLI dendrites. Furthermore, PF-PC synapses are encapsulated with glia, thus preventing neurotransmitter escape, whereas PF-MLI synapses lack ensheathing elements. As in the present work, the location of mGluR1α on PF-PC synapses was found to be perisynaptic (Baude et al., 1993; Lujan et al., 1996; Luján et al., 1997), and it was calculated that 50% of mGluR1α particles were located within 60 nm of the edge of the synapse. This percentage is close to the value reported here for MLIs. The close association of mGluR1s with the PSD rim in both synaptic types is consistent with the AMPAR/mGluR1 synergy proposed above. In addition, this spatial arrangement strongly suggests that in both cases, mGluR1 activation primarily results from homosynaptic glutamate release, and not from glutamate spillover. Nevertheless, differences between the two synaptic types are significant. The PSD size for PF-MLI synapses is twice smaller than that of PF-PC synapses (Abrahamsson et al., 2012), and this results in a significantly shorter distance between the site of vesicular release and mGluR1s, favoring homosynaptic activation of mGluR1s. On the other hand, while glial covering and glutamate uptake favor synapse isolation and hence homosynaptic signaling in PF-PC synapses, the absence of a diffusion barrier imposed by glial membranes around the PF-MLI synapse (Palay and Chan-Palay, 1974), and a lower density of glutamate transporter proteins (Chaudhry et al., 1995), would seem to favor activation by synaptic cross-talk in PF-MLI synapses. Nevertheless, the present finding of a close association of mGluR1 to the PSD at PF-MLI synapses suggests that the advantage of the short distance overrules the disadvantage of the lack of glial covering, so that overall, homosynaptic activation prevails also in the case of the PF-MLI synapse.

### Temporal pattern for mGluR1 activation

Compared to AMPARs, mGluR1s display slow activation kinetics and low desensitization rates. Consequently, single synaptic release results in a small number of activated receptors. A few pulses of high frequency release, however, can recruit a sufficient number of mGluR1s to trigger a measurable response as observed in slice experiments for PF-PC synapses (Finch and Augustine, 1998; Takechi et al., 1998) and for PF-MLI synapses (Karakossian and Otis, 2004). Longer stimulation trains are expected to recruit an increasing proportion of mGluR1s as their activation develops further while AMPAR desensitization occurs. Our in vivo results are in line with this expectation, showing a growing contribution of mGluR1s to PF-induced Ca_i_ responses as a function of train duration. We start seeing mGluR1-mediated Ca_i_ rises for bursts of PF activity lasting about 100 ms, a duration commonly recorded in vivo during sensory input or behavior (Jörntell and Ekerot, 2006). Given the above slice results, and because our GCaMP-based signals do not have the same resolution as signals reported by organic Ca^2+^ dyes, it is probable that the actual threshold for activation of mGluR1 in vivo is somewhat lower than our 100 ms estimate.

## Materials and methods

Experimental procedures complied with animal care guidelines of the University Paris Descartes and the guidelines of the Animal Care and Use committee of University of Science and Technology of China. Experimental procedures were approved by the ‘Prefecture de Police’ (#A-750607) in agreement with the European Directive 86/609/EEC and by the ethical committee of the University Paris Descartes.

Estimation of sample size was according to published in vivo and in vitro work. Sample size for each experiment is indicated in the main text.

### GCaMP expression in the cerebellar cortex

4–5 weeks old female mice heterozygous for an allele driving cre recombinase under the control of the PV promoter (Hippenmeyer et al., 2005), *Pvalb-Cre* mice, were deeply anesthetized with intraperitoneal injection of ketamine (14.8 μg/g)/xylane (10 μg/g) and mounted in a stereotaxic frame. A midline sagittal incision exposed the cranium over the cerebellar vermis. At the site of injection (6 mm from Bregma and 0.6 mm lateral) a 0.5 mm burr hole was drilled and a 34-gauge stainless steel beveled needle (WPI-nanofil) was slowly descended 0.4 mm through a slit cut in the meninges. After a 2 min pause, the needle was retracted 50 μm and then 1.2 μl of AAV2/1.hSyn.Flex.GCaMP3.WPRE.SV40, AAV2/9.hSyn.Flex.GCaMP3.WPRE.SV40 (Tian et al., 2009) or AAV2/1.hSyn.Flex. GCaMP5G.WPRE.SV40 (Akerboom et al., 2012) were injected at a rate of 0.1 μl/min. Viral constructs were purchased from the University of Pennsylvania Vector Core service. Once completed, the needle was left in place an additional 10 min before being withdrawn, the scalp sutured, and the mouse kept under a warming lamp until recovered from the anesthesia before finally being returned to standard housing.

### GCaMP imaging in anesthetized mice

2 to 4 weeks after stereotaxic vector delivery, mice were prepared for imaging as described in detail previously (Franconville et al., 2011). The time of expression was chosen to avoid GCaMP overexpression (Tian et al., 2009; Zariwala et al., 2012). In some sections, as indicated in the text, data was pooled from mice expressing GCaMP3 and GCaMP5G, since these proteins have similar Ca^2+^ affinities and kinetic parameters (Akerboom et al., 2012). During imaging anesthesia was delivered via an intraperitoneal catheter and vital parameters were continuously monitored using a Pulse Oxymeter system (Starr Life Sciences, USA). 2-photon laser scanning imaging was performed with a custom-built set-up (details in Franconville et al., 2011). Excitation wavelength was 910 nm. Imaging depth ranged from 50 to 100 μm from the surface, thus excluding basket cells.

Extracellular electrical stimulation was delivered through a theta-glass pipette filled with a HEPES-buffered extracellular saline to which 20 μM Alexa 594 dye was added to aid visualization. The pipette was placed on the superficial molecular layer more than 150 μm from the site imaged to avoid direct stimulation of the neurons in that field. Ag-AgCl electrodes connected the pipette to an isolated pulse stimulator (AM-systems) delivering 200 μs long pulses at 30–80 V amplitude. Initial search for stimulation site and parameters was performed with the criteria that 10 pulses at 100 Hz elicited Ca_i_ rises whose trial-to-trial variability did not exceed 10%. The number of repetitions and stimulation strength were kept to a minimum in order to limit activity-dependent changes in AMPAR function (Cull-Candy et al., 2006).

Glutamate receptor antagonists GYKI 53655, 5,7-Dichlorokynurenic acid (DCK), CPCCOEt and IEM 1460 were purchased from Tocris or Accent Scientific. Drugs were added to the artificial cerebrospinal fluid solution (ACSF) on the pool bathing the cranial window, which had the following composition (in mM): 150 NaCl, 2.5 KCl, 1 MgCl_2_, 1.5 CaCl_2_, 10 Hepes, adjusted to a pH of 7.3 with 1 M NaOH solution).

### GCaMP imaging in slices

One to three weeks after stereotaxic injection, sagittal cerebellar slices were prepared in sucrose-containing saline as described previously (Franconville et al., 2011) or using a low Na^+^ saline designed for slicing of adult mice brain (Zhao et al., 2011) with the following composition (in mM): 93 N-methyl-D-glucamine, 2.5 KCl, 1.25 NaH_2_PO_4_, 25 NaHCO_3_, 0.5 CaCl_2_, 10 MgCl_2_, 20 Hepes, 5 Na ascorbate, 2 thiourea, 3 Na pyruvate and 25 glucose (HCl added to bring pH to 7.4). Slices were maintained for 30–40 min at 34°C in standard recording saline (in mM: 125 NaCl, 2.5 KCl, 1.25 NaH_2_PO_4_, 25 NaHCO_3_, 2 CaCl_2_, 1 MgCl_2_, and 10 glucose) prior to transfer to the recording set-up. 2PLS imaging was performed at 34°C using an excitation wavelength of 910 nm. Even though precautions were taken to optimize slice survival, a variable fraction of MLIs displayed nuclear fluorescence after slicing. At equivalent expression times, nuclear fluorescence was not observed in vivo, suggesting that changes in the subcellular distribution of the GCaMP protein are due to the slicing procedure. Such changes are indicative of deleterious effects of slicing and therefore emphasize the importance of in vivo studies. MLIs were studied only if their nucleus did not show any fluorescence. Imaging was performed at depths of 40 to 70 μm from the slice surface. Imaging and extracellular stimulation were as described in Collin et al. (2009) and references within.

### Glutamate photorelease in slices

For whole-cell recording (WCR) experiments, slices from mice aged 30–35 days were prepared as described above. Extracellular recording saline contained (in mM): 135 NaCl, 4 KCl, 2 NaHCO_3_, 25 glucose, 2 CaCl_2_, 0.1 MgCl_2_ and 10 HEPES, pH 7.4 with NaOH. WCR was performed in MLIs with an intracellular solution containing (in mM): 140 Cs Gluconate, 4 MgCl_2_, 10 HEPES-K, 0.4 Na-GTP, 4 Na-ATP and 100 μM spermine, pH 7.3 with CsOH. MNI-glutamate (0.9 mM) was added to the recording chamber and photolysis was achieved with 100 μs pulses from a 405 nm laser as described elsewhere (Rossi et al., 2008). Currents evoked by MNI photolysis were recorded at holding potentials ranging from −100 to 80 mV. To study effects of Cd^2+^ on glutamate uncaging-evoked Ca_i_ signals, we prepared slices from transgenic animals (PN30-45) issued from a cross of mice carrying the GCaMP6f transgene in the Igs7 locus (Jackson labs stock number 024107) and *Pvalb-Cre* mice. Expression pattern was similar to that obtained with viral injections.

### Experimental design and data analysis for anesthetized and in vitro imaging

In vivo experiments were usually performed 2–3 days in a row in a week depending on the availability of the transgenic mice and the success of viral injection. In each animal, all activated neurons within the focal plane were sampled. Biological replicates were the number of animals used in the experiments. Technical replicates were the number of repeated stimulations for each condition. Outliers were not excluded from the data pool.

Data were analyzed with custom-written routines in Igor Pro (Wavemetrics). Background fluorescence Fb(t) was estimated as the average fluorescence of 20–30 pixels with the lowest F value throughout all the frames of the sequence. A somatic ROI was defined by a polygon drawn along the contour of the ring-shaped somatic pre-stimulus fluorescence. F(t) was calculated as the time course of the averaged fluorescence from all pixels in a ROI. ΔF/Fo was computed as (F(t)-Fo)/(Fo-FB). Fo and FB are the averages of F(t) and Fb(t) over the pre-stimulus period respectively. Values for pooled data are given as mean ± s.e.m. When assessing drug effects, results from GCaMP3 and GCaMP5 were pooled together, because these two GECIs have similar affinities for Ca^2+^ (Akerboom et al., 2012). For statistical analysis, we applied non-parametric two-tailed Wilcoxon signed test to test the null hypothesis based on the fact that data points were from different cells and their peak calcium transients should not follow a normal distribution. All the statistical information is indicated in the main text or figure legends. Groups were considered significantly different for p<0.05.

### GCaMP imaging in behaving mice

#### Chronic window and head-plate surgery

Three weeks after stereotaxic injection, mice were prepared for imaging during locomotion by implanting a chronic window and fixing a head post. Before surgery (10–15 min), mice were injected with buprenorphine (0.35 mg/kg intraperitoneal) for pain reduction. Lidocaine was used as a local anesthetic. Surgeries were performed under isoflurane (1.5–2.0% by volume in air) to maintain a surgical plane of anesthesia as determined by non-responsiveness to toe pinch. Eye ointment was applied under anesthesia at the start of surgery. Animals were held in a stereotaxic apparatus using ear and nose bars to stabilize the head. Body temperature was set with a heating pad and controller using biofeedback from a rectal thermocouple.

For the surgical procedure, the area of the head over the cerebellum was first shaved and the underlying skin was treated repeatedly with ethanol (70%) and Betadine in alternation. The skin above the skull was removed and the opening of the periosteum as well as any membranous tissue were cleaned. Once the skull was dry, a cranial window was carefully drilled using a handheld high-speed micromotor drill (Foredom). The center of the craniotomoy was located ~2 mm from lambda on the midline targeting lobule IV/V of the cerebellar vermis. Skull removal was performed under application of an ACSF solution to minimize the risk of damaging the dura overlying the brain. After skull removal, the ACSF solution was used to clean any debris from the brain surface and to maintain the brain moist providing an interface to the chronic window. A chronic window was fabricated from a cover glass (#1, thickness 150 μm) laser cut to a small oval shape (2.5 mm x 2 mm) to better fit the cranial opening, and to feature two holes of ~500 μm diameter and 1.5 mm separation on the major axis for drug delivery. The window was placed over the cerebellar vermis lobule IV/V and kept in direct contact with the brain by lowering the window with the help of a needle mounted in the stereotaxic setup. The chronic window was fixed in place with dental cement (Super-Bond C and B). After setting of the dental cement, the bone surrounding the craniotomy was thinned with the micromotor drill to allow for better access with the 20x objective. Then a custom-made stainless steel head post with a central opening for the craniotomy was cemented in place with dental cement (Super-Bond C and B). The chronic window was covered with silicone for protective purposes after surgery. Mice were carefully monitored during surgical recovery, with all animals showing normal behavior including the absence of motor deficits. Mice were allowed to recover at least 2 days before proceeding to the locomotion and imaging experiments with ad libitum access to food and water.

#### Fluorescence imaging during forced locomotion in awake mice

A sketch of the imaging setup can be seen in Figure 3A. For our experimental recordings and manipulations, mice were manually placed in a purpose-built apparatus by attaching the surgically implanted head post to a rigid fixture. Mice were positioned with the tip of the nose ~5 mm above the spherical treadmill, and with an angle of ~10° between the tangent of the treadmill and the bottom of the lower jaw, a position which was inferred from free walking on the treadmill. The treadmill's surface was covered with a plastic mesh (square grid of 5 × 5 mm) allowing for easy grip. Mice could walk self-paced on the treadmill. 2-photon laser scanning imaging was performed in the medial part of lobule IV/V of the cerebellar vermis region with a custom-built setup (for details, see Franconville et al., 2011), which was extended with a resonant scanning mirror (Sutter Resscan-Gen). Imaging was performed in the molecular layer at depths of 50 to 100 μm from the surface and the field of view was chosen in order to maximize the number of simultaneously recorded MLI somata. Excitation wavelength of the laser (MaiTai, Spectra Physics) was set at 910 nm and in some cases to 820 nm to measure movement related fluorescent changes. Laser power out of the objective (20x, 1.0 NA, Olympus, XLUMPlanFl N) was typically <80 mW. The photodetector was a photomultiplier tube (Hamamatsu, H7422P-40MOD) placed proximal to the imaging plane to maximize photon collection. Sequences of images were acquired at 30 Hz and comprised a 400 × 400 μm field of view with a pixel size of 0.78 × 0.78 μm using the ScanImage 2015 software (Pologruto et al., 2003).

Imaging recordings lasted for 40 s and were repeated every 2–4 min. The speed and the duration of locomotion during the recordings was imposed in an attempt to minimize variations across recordings. The forced locomotion scheme was as follows: Recordings started with a 6 s long period of baseline during which the mouse was able to freely rotate the treadmill. At 6 s, a servo motor moved a continuous rotation servo in a gear attached to the axis of the treadmill. The continuous servo started to linearly accelerates (3.33 cm/s^2^) the wheel at 7 s until a maximal speed of 10 cm/s was reached at 10 s. This rotation speed was kept constant until 23 s at which point the motor linearly decelerated (−3.33 cm/s^2^) until standstill at 26 s and then moved out of the gear on the treadmill axis allowing for self-paced walking. The rotation of the treadmill was recorded with a rotary encoder (Pewatron, E7P-700–315-D-D-D-3) at 40 kHz with a standard DAQ acquisition board (National Instruments, PCIe 6323). Mice were monitored with infra-red (940 nm LED illumination) videography at 200 frames/s (camera: Dalsa Genie Nano, NANO-M800-NIR) during the entire recording sessions.

#### Pharmacology in behaving mice

After recording baseline fluorescence during forced locomotion, the craniotomy bath ACSF solution (see composition above) was exchanged with a ACSF containing additionally 100 (animal #1A, #3) or 200 μM CPCCOEt (animal #1, #2, #4). Two holes of ~500 μm diameter were drilled in the cover-slips prior to implantation to allow for drug access. To further improve drug access, the dura mater was pierced with a sharp glass pipette before the experiment under anesthesia (1.5% isoflurane in air). In a subset of experiments (4 out of 5 recordings), 50 μM Alexa 594 was added to the drug-containing ACSF to assess the dynamics of drug diffusion in the recorded brain area (﻿Figure 3—figure supplement 1). 50 μM Alexa 594 was added to bath ACSF *after* the locomotion recordings with CPCCOEt in animals #1A.

Fluorescence recordings during forced-locomotion before- and after drug application were performed in 4 mice in total. One of the animals was recorded on two different days (animal#1A, animal#1). The first recording of animal #1, animal#1A, was removed from all further analysis based on the lack of fast Alexa fluorescence increase (Figure 3—figure supplement 1). The four remaining recordings from four different animals (#1,#2,#3,#4) were treated independently in the analysis described below.

#### Calcium imaging processing

The pre-processing of all raw calcium imaging data – image registration and ROI detection – was done using Suite2p using default settings (Pachitariu et al., 2007). ROI identity was furthermore visually verified and corrected when necessary for all recordings, that is, only ROIs spatially restricted to MLI somata were included in subsequent analysis. Subsequent image analysis was implemented using custom routines written in the Python programming language. Image stacks recorded before and after drug application were aligned using rigid translation through Enhanced Correlation Coefficient Maximization (Evangelidis and Psarakis, 2008) implemented in the OpenCV package for Python. Alignment was necessary since the application of the drug required removal of the objective which involved microscope stage movements. ROIs were aligned according to this translation and were considered to comprise the same soma if the overlap between the ROI areas before and after drug application was at least 30% of the total combined ROI area, that is, 0.3 ≦*A*_overlap_/*A*_union_ with *A*_overlap_ = *A*_before drug_ ∩ *A*_after drug_ and *A*_union_ = *A*_before drug_∪ *A*_after drug_. The fluorescent traces of overlapping ROIs were then compared between the before and after drug condition.

Cellular fluorescence traces were first compensated for surrounding neuropil signal by subtracting a scaled-down version, that is, *F^i,j^* = *F^i,j^*_MLI_- 0.7 *F^i,j^*_Neuropil_ (*i* denotes all identified ROIs, and *j* refers to the recording number). Subsequently, fluorescence traces were normalized using the pre-motorization baseline period (< 5 s). Blocks of recordings were scanned for a recording *j = k* with the least active baseline period based on the rotary encoder recordings, that is, the recording *j = k* for which the animal showed the least amount of locomotor activity during the first 5 s. Fluorescence during that least active baseline, referred to as *F_rest_*, was averaged for each ROI following $F_{rest}^{i}={\left\langle F^{i,j=k}{\left(t\right)}_{least\;active\;baseline}\right\rangle }_{t\in \left[0,5\right]}$, where *i* denotes the index of the respective ROI and brackets <> indicate the temporal averaging. ROI fluorescence traces of a block of recordings *j* were then normalized using this baseline activity according to $\Delta F^{i,j}/F_{rest}^{i}=\left(F^{i,j}-F_{rest}^{i}\right)/F_{rest}^{i}$.

In order to study locomotion-linked fluorescence, we calculated the 75^th^ percentile of $\Delta F^{i,j}/F_{rest}^{i}$ during the maximal-speed motorization period t∈[10,25] s. When using the 75th percentile, the fluorescence values of the drops of fluorescence during locomotion (see Figure 3C) do not affect the analysis. Rather, the 75th percentile emphasizes the high fluorescence parts on which we want to focus here. We observed an overall decay of locomotion fluorescence with increasing number of repetitions, which was shared across all ROIs (see Figure 3E). In order to compensate for that decay, we fitted a single-exponentially decaying function $f\left(j\right)=A+Be^{-j/C}$ (where *j* denotes pre-drug recording numbers as linearly increasing integers; A, B, C are fit parameters) to the *pre-drug* locomotion fluorescence values. This function is subtracted from *all* recordings, pre- and after-drug, which normalized the pre-drug locomotion fluorescence to zero and revealed any drug induced change beyond the general decay (see Figure 3F). Note that by considering the decay as function of recordings *j*, the fluorescence decay is linked to the increasing number of the recordings rather than explicit time.

To test for changes in drug-induced fluorescence, we performed paired t-test comparisons for each ROI between all pre- and post-drug locomotion fluorescence values (see Figure 3) excluding the first three recordings after drug-delivery to account for the wash-in time of the drug (τ = 5.5 min, see Figure 3—figure supplement 1).

To assess fluorescence changes linked to calcium vs. changes related to movement artifacts in the recorded tissue, we performed forced locomotion recordings before application of CPCCOEt at 820 nm laser excitation wavelength at which GCaMP fluorescence is insensitive to the calcium changes (see Figure 3—figure supplement 2). The calcium imaging recordings went through the same image processing procedure as outlined above for the 910 nm excitation wavelength recordings. However, ROI detection was impaired in Suite2p due to the strongly reduced temporal ROI fluorescence dynamics. ROIs corresponding to MLI cell bodies were therefore mostly labeled by hand using oval shapes. Fluorescence images from before drug application and 820 nm excitation runs were aligned (see above). ROIs with an overlap of at least 30% of the total ROI area were considered corresponding to the same cell body (see above). The standard deviation and 75^th^ percentile of the $\Delta F^{i,j}/F_{rest}^{i}$ were calculated for the maximal-speed locomotion period ([10,25] s). The same values were extracted for the corresponding ROIs from the runs before drug application and averaged. Figure 3 summarizes the ratios between the 820 and the 910 nm excitation wavelength runs of all ROIs per animal. A fraction of 30–50% of the fluorescence changes can be attributed to movement artifacts (Figure 3—figure supplement 2A), while the increase in fluorescence is largely driven by calcium (Figure 3—figure supplement 2B). The calcium-associated increase in fluorescence is the focus of the here presented analysis.

All analysis scripts are available online (Graupner, 2020; copy archived at https://github.com/elifesciences-publications/mGluR_MLI_in-vivo).

### Immunocytochemistry

Mice were anesthetized by intraperitoneal injection of sodium pentobarbital (Roche) and transcardially perfused by cold Phosphate Buffer Saline (PBS) followed by PBS containing 4% of paraformaldehyde (PFA). After decapitation the cerebellum was removed and post fixed in PBS PFA solution for 2 hr. 50 μm thick parasagittal slices were prepared from the vermis using a vibrating slicer (Leica VT 1000S, Leica Microsystems, Germany). The slices were first incubated in PBS containing 0.3% Triton and 10% fetal bovine serum for 5 hr at room temperature then at 4°C overnight with a mixture of rabbit polyclonal serum anti-Parvalbumin (PV25, SWANT, Marly, Switzerland) and guinea pig polyclonal serum anti calbindin D28k (Synaptic Systems, Gôttingen, Germany) diluted to 1/1000 in PBS containing 1 mg/ml of Bovine Serum Albumine (BSA). After 3 washes in PBS, the slices were incubated for 3 hr with secondary goat antibodies anti-rabbit IgG and anti-guinea pig IgG conjugated to Alexa fluor 546 and Alexa fluor 647 respectively (1/500, Molecular Probes, Eugene, OR). Slices were mounted between slides and cover slip with Vectashield Mounting Medium (Vector lab, Burlingame, CA). Stack of images (0.44 µm thickness) were acquired using an LSM 510 confocal microscope (Zeiss, Jena, Germany) equipped with Plan-Neofluar 40x/1.3 oil objective and appropriate excitation (488, 543 and 633 nm) and emission filters (BP 505–530, BP560_615 and LP 650). Image analysis and projections were done by LSM and ImageJ Software.

### SDS-digested freeze-fracture replica labeling (SDS-FRL)

SDS-FRL was performed as described previously (Masugi-Tokita et al., 2007). Adult Wistar rats were perfused transcardially with 2% paraformaldehyde (PFA) and 15% saturated picric acid solution in 0.1 M phosphate buffer (PB). Sagittal slices (130 μm thick) were cut from middle cerebellar lobules and trimmed slices containing molecular layer were rapidly frozen by a high-pressure freezing machine (HPM010, BAL-TEC, Balzers), fractured into two parts at −140°C, and replicated by a freeze-fracture replica machine (JFD II, JEOL, Tokyo). Tissue debris was dissolved with gentle shaking at 80°C for 18 hr in a solution containing 15 mM Tris-HCl (pH 8.3), 20% sucrose, and 2.5% SDS. The replicas were reacted with rabbit anti-mGluR1α antibody (Shigemoto et al., 1997) (1:500 dilution) followed by anti-rabbit secondary antibody conjugated with 10 nm gold particles (British Biocell International, BBI, Cardiff). The specificity of freeze-fracture replica labeling for mGluR1 was verified using mGluR1 KO mice, as shown in Mansouri et al., 2015. The labeled replicas were examined using a transmission electron microscope (JEM 1010, JEOL, Tokyo).

## Data Availability

All data generated or analysed during this study are included in the manuscript and supporting files.

## Additional files

Transparent reporting form
