# Supplementary material for: Synergism of type 1 metabotropic and ionotropic glutamate receptors in cerebellar molecular layer interneurons in vivo
Source: eLife. 2020 May 13;9:e56839. doi: 10.7554/eLife.56839 (PMC7220378; doi:10.7554/eLife.56839)
Supplement: Peer review history

# Decision letter

**DOI:** 10.7554/eLife.56839.sa1

**Affiliations:** RIKENJapan; Aarhus UniversityDenmark

In the interests of transparency, eLife publishes the most substantive revision requests and the accompanying author responses.

**Acceptance summary:**

Bao et al. examined whether type 1 metabotropic glutamate receptors (mGluR1s) are engaged at the parallel fiber-molecular layer interneuron synapses in the cerebellum during physiological activity in awake behaving mice. Through a combination of in vivo calcium imaging, slice electrophysiology and freeze-fracture electron microscopy, the authors demonstrate the recruitment of mGluR1-dependent calcium signals during locomotion, and that mGluR1 activation is synergistic with the activation of ionotropic glutamate receptors to produce the rise in intracellular calcium.

**Decision letter after peer review:**

Thank you for submitting your work entitled "Synergistic action of metabotropic and ionotropic glutamate receptors in cerebellar molecular layer interneurons in vivo" for consideration by *eLife*. Your article has been reviewed by three peer reviewers, one of whom is a member of our Board of Reviewing Editors, and the evaluation has been overseen by a Senior Editor. The following individuals involved in review of your submission have agreed to reveal their identity: Marco Capogna (Reviewer #2).

All three reviewers agree that the study is of high potential interest. However, major issues have been raised such that the conclusions are not firmly supported by the data shown. Because it is *eLife*'s policy to reject manuscripts that are likely to require more than 2 months of additional work, the decision was made to reject this version so that you are free to submit it elsewhere at this time, should you wish. Nonetheless, all the reviewers and editors see the very high potential value and interest of the work, and if you agree that doing the requested experiments will strengthen the manuscript, *eLife* would welcome a new submission of this work. The authors are encouraged to take the time to address the concerns thoroughly.

Reviewer #1:

The study addresses physiological requirements for activation of group 1 mGluRs and their functional and spatial relationship to AMPARs at the parallel fiber-molecular layer interneuron (MLI) synapses in the cerebellum in vivo. Using GCaMP Ca imaging of MLI neurons, the authors report that longer duration PF stimulation (1 s rather than 200 ms) at 100 Hz is more effective in eliciting mGluR1-dependent Ca rises in MLI soma, in a manner that is synergistic with activation of AMPARs. Interestingly, AMPARs involved are not Ca-permeable in contrast to previous studies at developing MLI synapses, and the depolarization caused by AMPAR activation likely recruits VGCC to confer VGCC-dependence of mGluR1 activation as shown in acute slices. The authors also perform ultrastructural analysis of mGluR1 subcellular distribution by freeze-fracture replica labelling, which shows its localization close to the PSD. Altogether, in identifying several features of mGluR1 involvement at PF-MLI synapses in vivo, while somewhat descriptive, the findings are valuable. The major technical issue is the use of different GCaMP variants. While the initial demonstration of sensory stimuli-evoked MLI activity and its similarity to PF stimulation-triggered response are shown by GCaMP6s, subsequent analyses use mostly GCaMP3 imaging which not only differ in sensitivity for detecting MLI activity but also display different kinetics. In addition, demonstration of the involvement of mGluR1 for 100 ms-long trains at 500 Hz is shown using GCaMP5. There needs to be a consistency in the activity readout used across experiments to compellingly demonstrate that mGluR1 activity as reported is involved in vivo under physiologically relevant paradigms.

Reviewer #2:

This work directed by Isabel Llano and Ryuchi Shigemoto investigates parallel fibers (PFs) glutamatergic synapses onto molecular layer interneurons (MLI) of the mouse cerebellum. They found that mGluR1s are activated in vivo by synaptically-released glutamate and act together with ionotropic (i)GluRs to trigger intracellular calcium rise. Furthermore, and this is one of the nicest findings of this manuscript, high resolution analysis of the spatial distribution of postsynaptic mGluR1 PF-MLI synapse distribution show that these receptors are remarkable close to iGluRs, consistent with their observed synergic functional effects.

The manuscript is competently written and illustrated. The senior authors are expert researchers in the field of cerebellum synapses, calcium imaging and high resolution receptor localization analysis.

Overall, I like this manuscript. I have some constructive criticism that I hope will help the Authors to improve the quality and completeness of their study and ultimately enhance the impact of their manuscript when it will be published.

1) It is a pity that most of the study uses electrical stimulation of PFs, and not a more physiological way to stimulate the PFs-MLI pathway, to investigate the calcium signaling in MLIs and iGluRs/mGluR1 synergic activation. In a few experiments, calcium responses of MLI are evoked by air puffs. However, due to the relatively high calcium signal variability observed after air puff stimulation, the Authors decided to conduct most of their pharmacological experiments by using electrical stimulation. On the contrary, I recommend the Authors to reproduce key results (eg, iGluRs/mGluR1 synergic activation) by using air puffs stimulation, and to include the results of these new experiments in the revised version of the manuscript. Moreover, it would be extremely interesting to investigate the PFs-MLI synapses and the iGluRs/mGluR1 signaling evoked by eyeblink conditioning, a form of classical conditioned learning, that overwhelming evidence demonstrate to involve cerebellar synapses as major substrate. In my opinion to include the requested new experiments (more air puff data and eyeblink conditioning) would enhance very significantly the significance and the impact of this work.

2) Drugs are applied to the pool bathing the craniotomy and no wash out is possible and observed. Furthermore, the actual concentration of the drugs reaching the receptors remains unknown. It would useful to perform some control experiment (e.g., application of a drug at two or three different concentrations; or fluorescence imaging of applied drug diffusion) to be reassured that the lack of the action of some drugs (e.g., IEM1460) is a genuine result and not (at least in part) due to poor drug diffusion to plasma membranes containing AMPARs.

Reviewer #3:

The manuscript by Bao et al. seeks to find out whether mGluRs are engaged on Molecular Layer Interneurons (MLIs) of the Cerebellum during physiologically relevant synaptic activation in vivo and whether their signalling is dependent on iGluR activity. To answer these questions, the authors perform novel and technically demanding recordings of Ca^2+^ transients from MLIs in vivo, and also in ex vivo slices. These are important questions and innovative techniques are employed to address them, but the data are preliminary which leaves the conclusions poorly supported.

Major comments:

1) It is claimed that MLIs are activated as a "beam" by air puffs. This is not quantified so conclusions on this question are premature. This also questions whether it is reasonable to replicate the physiological stimulation with electrical stimulation of the PFs.

2) The observed timecourse of the putative mGluR1-mediated calcium response is very rapid for a G-protein induced response (eg Figure 2). Furthermore, the timecourse seems to be exactly the same for both mGluR1-mediated and iGluR-mediated components. This doesn't seem likely which calls into question whether the responses are indeed mediated by mGluR1. To confirm this requires more than just a single drug application, which could in fact be inhibiting glutamate release from PFs.

3) The rationale for the use of direct PF stimulation is that it gives a more reliable signal than air puffs and that they are equivalent. However, the signals are not that similar despite the assertion. Decay rates much slower for air puffs. Furthermore the most equivalent stimulation is 20 pulses at 100Hz but at this stimulation duration and frequency there is little effect of mGluR1.

4) One of the core findings claimed in the manuscript is the synergy between iGluR and mGluR but this is never directly demonstrated. Need to compare application of iGluR antagonist and then mGluR antagonist vs mGluR antagonist alone and quantify the difference.

5) Overall there is reasonable evidence for lack of Ca-permeable AMPARs but it would be useful to show a positive control for IEM use in vivo. What does it do in vitro?

6) The experiment to test role of VGCCs is very preliminary. Photorelease of Glu onto the soma likely initiates APs leading to the observed calcium signal. The fact this is blocked in a single experiment is not convincing and in any case does not reveal much about whether the AMPARs are calcium permeable. The claim that "response of MLIs to mGluR1 agonists is abolished by inclusion of VGCC blocker Cd2+" is not supported by the data.

7) An alternative possibility for synergy between iGluR and mGluR is electrotonic effects on glutamate diffusion (Sylantyev et al., 2013). However, insufficient evidence is presented to distinguish between this and Ca^2+^-dependent mechanisms.

[Editors’ note: further revisions were suggested prior to acceptance, as described below.]

Thank you for resubmitting your article "Synergism of type1 metabotropic and ionotropic glutamate receptors in cerebellar molecular layer interneurons in vivo" for consideration by *eLife*. Your article has been reviewed by three peer reviewers, one of whom is a member of our Board of Reviewing Editors, and the evaluation has been overseen by Gary Westbrook as the Senior Editor The following individual involved in review of your submission has agreed to reveal their identity: Marco Capogna (Reviewer #2). The reviewers have discussed the reviews with one another and the Reviewing Editor has drafted this decision to help you prepare a revised submission.

We would like to draw your attention to changes in our revision policy that we have made in response to COVID-19 (https://elifesciences.org/articles/57162). Specifically, we are asking editors to accept without delay manuscripts, like yours, that they judge can stand as *eLife* papers without additional data, even if they feel that additional data would make the manuscript stronger.

Summary:

This is a revision of a previously submitted study, examining the activation of group 1 mGluRs at parallel fiber-molecular layer interneuron (MLI) synapses in the cerebellum during physiological activity, now monitored in awake behaving mice. The authors demonstrate recruitment of mGluR-dependent Ca signals during locomotion, and that mGluR activation is synergistic with the activation of iGluRs.

Although the two reviewers of the original submission found that the revision suitably addressed their points, the third reviewer who was new, had some concerns, especially pointing out that although demonstrating the activation of mGluRs during walking was novel, whether this resulted from PF activity similar to the electrical stimulation used in anesthetized mice and in other slice physiology experiments remained unclear. Upon discussion amongst all three reviewers and the senior editor, we request that the authors carefully address the following points by making use of data or information that they might already possess, by toning down the claims or by referencing existing literature.

1) Information about the intensity of the electrical stimulation used in anesthetized mice experiments and how it relates to the data acquired in awake behaving mice would be valuable. That is, how many PFs are being recruited? What EPSC amplitudes are associated with the reported calcium transients?

2) Reword the text inciting anticipation that the study addresses a specific role for mGluRs in synaptic plasticity, for example at the start of the Abstract.

3) The freeze-fracture EM data is very nice and convincing. However, many of the measurements in the manuscript are taken from the cell body where surely few excitatory synapses are found. The authors should acknowledge this issue in the text.

4) It would be nice to have more details about the location of the imaging, i.e. how deep into the molecular layer. Having this information would help the reader determine the type of cells imaged because there could be functional heterogeneity between cells localized in different parts of the molecular layer.

5) Figure 2D and 2E. If the bar at the very left in both panels represent the same dataset, this should be stated in the legend.

# Reply: Author response

**DOI:** 10.7554/eLife.56839.sa2

[Editors’ note: the authors resubmitted a revised version of the paper for consideration. What follows is the authors’ response to the first round of review.]

Reviewer #1:

[…] The authors also perform ultrastructural analysis of mGluR1 subcellular distribution by freeze-fracture replica labelling, which shows its localization close to the PSD. Altogether, in identifying several features of mGluR1 involvement at PF-MLI synapses in vivo, while somewhat descriptive, the findings are valuable. The major technical issue is the use of different GCaMP variants. While the initial demonstration of sensory stimuli-evoked MLI activity and its similarity to PF stimulation-triggered response are shown by GCaMP6s, subsequent analyses use mostly GCaMP3 imaging which not only differ in sensitivity for detecting MLI activity but also display different kinetics. In addition, demonstration of the involvement of mGluR1 for 100 ms-long trains at 500 Hz is shown using GCaMP5. There needs to be a consistency in the activity readout used across experiments to compellingly demonstrate that mGluR1 activity as reported is involved in vivo under physiologically relevant paradigms.

We thank this reviewer for her/his appreciating the value of our work. The main objection raised by this review was that “While the initial demonstration of sensory stimuli-evoked MLI activity and its similarity to PF stimulation-triggered response are shown by GCaMP6s, subsequent analyses use mostly GCaMP3 imaging which not only differ in sensitivity for detecting MLI activity but also display different kinetics.” To address this criticism, we have removed data using GCaMP6 from this ms. The new set of experiments in behaving mice (Figure 3 and Figure 3—figure supplement 1 and 2) were performed in animals expressing GCaMP3, that is, the same protein as that used for the initial characterization of the effect of mGluR block on PF-evoked Cai rises. These new experiments demonstrate that mGluRs are activated in awake and physiologically relevant conditions.

Reviewer #2:

[…] Overall, I like this manuscript. I have some constructive criticism that I hope will help the Authors to improve the quality and completeness of their study and ultimately enhance the impact of their manuscript when it will be published.

1) It is a pity that most of the study uses electrical stimulation of PFs, and not a more physiological way to stimulate the PFs-MLI pathway, to investigate the calcium signaling in MLIs and iGluRs/mGluR1 synergic activation. In a few experiments, calcium responses of MLI are evoked by air puffs. However, due to the relatively high calcium signal variability observed after air puff stimulation, the Authors decided to conduct most of their pharmacological experiments by using electrical stimulation. On the contrary, I recommend the Authors to reproduce key results (eg, iGluRs/mGluR1 synergic activation) by using air puffs stimulation, and to include the results of these new experiments in the revised version of the manuscript. Moreover, it would be extremely interesting to investigate the PFs-MLI synapses and the iGluRs/mGluR1 signaling evoked by eyeblink conditioning, a form of classical conditioned learning, that overwhelming evidence demonstrate to involve cerebellar synapses as major substrate. In my opinion to include the requested new experiments (more air puff data and eyeblink conditioning) would enhance very significantly the significance and the impact of this work.

To deal with this issue, we searched for an experimental paradigm that allows us to perform 2-photon calcium imaging in behaving mice. While the suggestion of the reviewer to study signals “evoked by eyeblink conditioning” is interesting, we preferred to continue working in the same cerebellar region as that used during the studies with electrical stimulation of PFs. This choice is dictated by the need to decrease biological variability, already large in a behaving situation. We decided to a conduct pharmacological study on the effect of mGluR1 block on the calcium signals recorded from MLIs while mice walk in a wheel since walking was previously shown to engage MLI activity in the vermis both with electrophysiological recordings (Jelitai et al., 2016) and with calcium imaging (Ozden et al., 2012). The results of these experiments are described in section “Activation of mGluRs in MLIs during locomotion” (Figure 3 and Figure 3—figure supplement 1 and 2).

The data show that mGluR1 block decreases the magnitude of the calcium signals associated with walking. Furthermore, the amount of inhibition depends on the initial amplitude of the signal, which is in accord with the results obtained with electrical stimulation of PFs.

2) Drugs are applied to the pool bathing the craniotomy and no wash out is possible and observed. Furthermore, the actual concentration of the drugs reaching the receptors remains unknown. It would useful to perform some control experiment (e.g., application of a drug at two or three different concentrations; or fluorescence imaging of applied drug diffusion) to be reassured that the lack of the action of some drugs (e.g., IEM1460) is a genuine result and not (at least in part) due to poor drug diffusion to plasma membranes containing AMPARs.

It is true that in vivo recordings do not offer the same level of quantification as does slice work. The situation is not well suited to perform dose-response curves. However, we do have several lines of evidence indicating that the drugs reach the molecular layer at sufficiently high concentrations to achieve the desired block. Concerning experiments on anesthetized mice, as shown in Figure 2, addition of ionotropic blocker after mGluR block totally abolishes the calcium signals elected by PF stimulation.

Concerning the new experiments in behaving mice, we added Alexa 594 to the craniotomy and followed the time course of fluorescence increase. The results are shown in Figure 3—figure supplement 1 and indicate that the Alexa concentration equilibrates with a time constant of 5 minutes. Since Alexa and CPCCOEt have similar molecular weights, the time constant of equilibration of CPCCOEt is likely close to 5 minutes as well.

Reviewer #3:

The manuscript by Bao et al. seeks to find out whether mGluRs are engaged on Molecular Layer Interneurons (MLIs) of the Cerebellum during physiologically relevant synaptic activation in vivo and whether their signalling is dependent on iGluR activity. To answer these questions, the authors perform novel and technically demanding recordings of Ca2+ transients from MLIs in vivo, and also in ex vivo slices. These are important questions and innovative techniques are employed to address them, but the data are preliminary which leaves the conclusions poorly supported.

We thank the reviewer for her/his review of our work. The general objection of the reviewer is that data is preliminary: “, but the data are preliminary which leaves the conclusions poorly supported.” This statement was primarily directed to the air puff experiments, which have been removed. For the rest of the manuscript, we stress that our initial presentation of our results was very succinct, thus giving perhaps a false impression of superficiality. In fact our work, as presented in the original manuscript, strongly supports the conclusion that mGluRs type 1 are engaged in MLIs following PF stimulation in anesthetized mice. The new experiments that have now been incorporated to the revised version show that these receptors are also recruited during walking in awake mice. Furthermore, the manuscript investigates details of iGluR-mGluR interactions, and it presents for the first time the distribution of mGluR1 at the PF-MLI synapse, using quantitative EM. Altogether, the work presents a complete description of the mGluR1 component of PF-MLI synapses, all the way from the molecular level up to awake in vivo data. In the revised version, we have added some quantification and experimental detail to document better the experimental bases of our conclusions.

Major comments:

1) It is claimed that MLIs are activated as a "beam" by air puffs. This is not quantified so conclusions on this question are premature. This also questions whether it is reasonable to replicate the physiological stimulation with electrical stimulation of the PFs.

This data set has been removed.

2) The observed timecourse of the putative mGluR1-mediated calcium response is very rapid for a G-protein induced response (eg Figure 2). Furthermore, the timecourse seems to be exactly the same for both mGluR1-mediated and iGluR-mediated components. This doesn't seem likely which calls into question whether the responses are indeed mediated by mGluR1. To confirm this requires more than just a single drug application, which could in fact be inhibiting glutamate release from PFs.

Concerning the speed of the responses: G-protein induced responses vary considerably in their onset kinetics. Our own work on MLIs has shown that mGluR1-induced currents and mGluR1- induced calcium rises in these cells can rise within a few tens of ms after application of a glutamatergic agonist (Collin et al., 2005). Such a time course is compatible with the time course of calcium rises observed in the present work.

Concerning the comparison of response kinetics with and without blockers: Because we are using a calcium probe with relatively slow kinetics, the time course of the calcium rises is largely dictated by the probe and is unlikely to give us insight into the receptors and/or signaling pathways involved. This is the reason that we have refrained from time course considerations.

Concerning the drug application, which could in fact be inhibiting glutamate release from PFs.:We are not aware of any evidence for mGluR1 being expressed at the presynaptic side of central synapses. R Shigemoto’s laboratory has looked at different stages of development and found no immunoreactivity for mGluR1 at PF axons (Lopez-Bendito et al., 2001). On the other hand, block of mGluR1 reduces the LTD induced at PF-stellate cell synapses with certain stimulation protocols (Soler-Llavina and Sabatini, 2006). As endocannabinoid production in PCs is enhanced by mGluR1 activation, the LTD is thought to involve an inhibition of glutamate release by endocannabinoids receptors present in PFs. Although we have no evidence for LTD induction in our work, one could argue that there is an endogenous level of LTD at this synapse that is modulated by endocannabinoids. However, block of mGluR1 would be expected to decrease endocannabinoid production and then increase glutamate release. Therefore, this mechanism has the wrong sign to explain the observed inhibition associated with mGluR1 block.

3) The rationale for the use of direct PF stimulation is that it gives a more reliable signal than air puffs and that they are equivalent. However, the signals are not that similar despite the assertion. Decay rates much slower for air puffs. Furthermore the most equivalent stimulation is 20 pulses at 100Hz but at this stimulation duration and frequency there is little effect of mGluR1.

The comparison does not longer apply, as the data set from air puff experiments has been removed.

4) One of the core findings claimed in the manuscript is the synergy between iGluR and mGluR but this is never directly demonstrated. Need to compare application of iGluR antagonist and then mGluR antagonist vs mGluR antagonist alone and quantify the difference.

We agree that our rationale was not explained correctly and we added an explicit comparison for the percentage of block observed with iGluRs versus mGluR1 antagonists. Concerning the suggestion of blocking mGluR1 in the presence of iGluRs antagonists, as shown in Figure 4B, blocking iGluRs results in an almost total block of the calcium rises evoked by PF stimulation (>90 % block at 100 Hz during 1 s), so that it is impractical to study the pharmacology of the remaining response. By contrast, applying CPCCOEt alone inhibits the response by approximately 50 % (Figure 2D), and the remaining response is abolished by iGluR blockers (Figure 2C).

5) Overall there is reasonable evidence for lack of Ca-permeable AMPARs but it would be useful to show a positive control for IEM use in vivo. What does it do in vitro?

We have no positive control for this drug in vivo. However, the fact that calcium rises are abolished after addition of mGluR1 and iGluRs blockers (Figure 2) indicates that drugs do reach the molecular layer. Furthermore, the new data set with ALEXA (Figure 3—figure supplement 1) gives us a time constant of 5 minutes for diffusion of this fluorophore in the tissue. These results suggest that IEM, as well as the other drugs used, did reach the imaged site. And given the published effect of IEM in vitro , the absence of effect on the calcium rises can be interpreted as indicative of lack of calcium-permeable AMPA Rs.

6) The experiment to test role of VGCCs is very preliminary. Photorelease of Glu onto the soma likely initiates APs leading to the observed calcium signal. The fact this is blocked in a single experiment is not convincing and in any case does not reveal much about whether the AMPARs are calcium permeable. The claim that "response of MLIs to mGluR1 agonists is abolished by inclusion of VGCC blocker Cd2+" is not supported by the data.

We conducted new experiments in slices to verify that the signals evoked by 1ms glutamate uncaging over the soma are due to mGluR1 activation. The results were clear, with robust response in control saline and hardy any signal in the presence of the mGluR1 blocker. This is now reported in subsection “Synergy between iGluRs and mGluR1s is not mediated by Ca^2+^-permeable AMPARs”.

Concerning the block by Cd, the block shown in Figure 4C is one example that is representative of 4 experiments as stated in the text (subsection “Synergy between iGluRs and mGluR1s is not mediated by Ca^2+-^permeable AMPARs”).

7) An alternative possibility for synergy between iGluR and mGluR is electrotonic effects on glutamate diffusion (Sylantyev et al., 2013). However, insufficient evidence is presented to distinguish between this and Ca2+-dependent mechanisms.

We added a paragraph to discuss this possibility (subsection “Synergistic Ca_i_ rises following combined activation of mGluR1s and iGluRs”).

[Editors’ note: what follows is the authors’ response to the second round of review.]

Although the two reviewers of the original submission found that the revision suitably addressed their points, the third reviewer who was new, had some concerns, especially pointing out that although demonstrating the activation of mGluRs during walking was novel, whether this resulted from PF activity similar to the electrical stimulation used in anesthetized mice and in other slice physiology experiments remained unclear. Upon discussion amongst all three reviewers and the senior editor, we request that the authors carefully address the following points by making use of data or information that they might already possess, by toning down the claims or by referencing existing literature.

1) Information about the intensity of the electrical stimulation used in anesthetized mice experiments and how it relates to the data acquired in awake behaving mice would be valuable. That is, how many PFs are being recruited? What EPSC amplitudes are associated with the reported calcium transients?

The intensity of the extracellular stimulation (30 to 80 V; 200 µs long pulses) as well as the criteria for choosing the stimulation voltage are described in Materials and methods as:

“The pipette was placed on the superficial molecular layer more than 150 µm from site imaged to avoid direct stimulation of the neurons in that field. Ag-AgCl electrodes connected the pipette to an isolated pulse stimulator (AM-systems) delivering 200 µs long pulses at 30-80 V amplitude. Initial search for stimulation site and parameters was performed with the criteria that 10 pulses at 100 Hz elicited Ca_i_ rises whose trial-totrial variability did not exceed 10 %.”

To estimate how comparable are signals recorded with parallel fiber (PF) stimulation protocol and those during locomotion, we first compared the calcium transients evoked in these two types of experiments. For 0.2 to 1 sec long PF trains at 100 Hz, we obtained peak DF/Fo of 114+31 and 347+43 % (mean+sem) respectively, whereas for the locomotion associated transients the corresponding values were 128.2+8.5 %. Thus, calcium transients in MLIs somata are in a comparable range for both experimental conditions. Secondly, as the reviewers suggested, we can estimate the EPSC in MLIs under these two conditions. We have data from Jin Bao’s paper in Journal of Neuroscience (2010) reporting in stellate cells an EPSC amplitude of ~100 pA following stimulation of GCs with theta glass pipettes, as used in the current study. This value is comparable to that obtained from in vivo patch clamp recordings of stellate cells in locomoting mice (60-70 pA, Supl. Figure 3, Jelitai, et al., 2016). However, spatial and temporal patterns of activation differ. The electrical stimulation we use imposes a “beam” like pattern of MLI recruitment (with a width in the range of 20 to 50 µm; Figure 1, see also Astorga et al., 2015). On the other hand, locomotion engages a much larger population of MLIs (Figure 3) in accord with results from Ozden et al., 2012. Temporal differences are also expected, as discussed below.

Concerning the number of PFs recruited: In extracellular stimulation experiments, we presume that action potential thresholds are rather homogeneous across PFs, so that all PFs within a beam are excited at the frequency imposed by extracellular stimulation. During locomotion, likewise, a vast majority of GCs are activated (94% of all GCs in a large field of view, according to Ozden et al., 2012). The impact on MLIs is a change in firing rate from an average value of 20 Hz in quiet mice to 60 Hz during self-paced locomotion (Jelitai et al., 2016). GC firing in mice walking spontaneously is very heterogeneous in time and in space. In time, firing occurs in high frequency bursts with frequency within a burst ranging from 60 to 142 Hz, and burst durations ranging from 30 to 140 ms. Strikingly, these values are close to those needed to obtain measurable mGluR responses with extracellular stimulation (Figure 2). In space, individual GCs are reported to vary markedly in their firing patterns when mice are walking. In view of the results of Figure 3 indicating a measurable mGluR contribution in a rather small proportion of MLIs, it is plausible that particularly active GCs cooperate within the dendritic field of these particular MLIs to produce mGluR activation as a result of temporal and spatial summation of the perisynaptic glutamate concentration.

We added a slightly shorter version of these arguments to Discussion.

2) Reword the text inciting anticipation that the study addresses a specific role for mGluRs in synaptic plasticity, for example at the start of the Abstract.

We deleted from the Abstract the mention of synaptic plasticity. We also removed from Introduction the sentence referring to “other forms of synaptic plasticity”.

3) The freeze-fracture EM data is very nice and convincing. However, many of the measurements in the manuscript are taken from the cell body where surely few excitatory synapses are found. The authors should acknowledge this issue in the text.

We acknowledge now this difference in the context of presenting the results of mGluR block on the neuropil as follows:

“Nonetheless, analysis of the neuropil allows us to confirm that mGluRs are engaged in dendrites, where the density of parallel fibers synapses is 3 fold higher than in the soma (Abrahamsson et al., 2012).”

4) It would be nice to have more details about the location of the imaging, i.e. how deep into the molecular layer. Having this information would help the reader determine the type of cells imaged because there could be functional heterogeneity between cells localized in different parts of the molecular layer.

Imaging depth ranged from 50 to 100 µm from the surface (now specified in the Materials and methods). In both experimental paradigms we imaged at depths at which the MLI population will comprise stellate cells.

5) Figure 2D and 2E. If the bar at the very left in both panels represent the same dataset, this should be stated in the legend.

Added to Figure 2E legend: “The 100 Hz, 0.1 s bin is derived from the same data set as that used for the first bin in panel D.”
